# Supplementary material for: Barriers and facilitators to healthy lifestyle and acceptability of a dietary and physical activity intervention among African Caribbean prostate cancer survivors in the UK: a qualitative study
Source: BMJ Open. 2017 Oct 15;7(10):e017217. doi: 10.1136/bmjopen-2017-017217 (PMC5652511; doi:10.1136/bmjopen-2017-017217)
Supplement: Supplementary file 2 [file bmjopen-2017-017217supp002.pdf]

Supplementary Material 1

Diet and Lifestyle after Prostate Cancer Diagnosis

Topic Guide

Aims and Objectives

The aim of this study is to gather the evidence for dietary and lifestyle changes in prostate cancer survivors to inform a dietary and lifestyle intervention for randomised trials. This will involve exploring:

- Motivators and barriers to changes in dietary and lifestyle behaviours
- Sources of information on diet and lifestyle
- Experience and views of dietary and lifestyle interventions
- Preferences for intervention (delivery format)

Introduction

- Thank you for participating
- Introduce self and Uni Bristol
- Introduce the study: what is it about and why (limited information)
- Talk through key points:
  - length of interview
  - interview like a discussion, but will cover key topics
  - no right or wrong answers
  - participation is voluntary, rights to withdraw
  - recording interview (concentrate on what you are saying, accuracy)
- Confidentiality and anonymity, how findings will be reported
- Thank you payment
- Questions?
- Happy to proceed? Sign consent form

START RECORDING

1. Background and medical history

- **Age; household** (live alone or with others)
  - relationship with
  - married/widowed/single/partner

- **Place of birth; religion**
  - if born outside of UK, where? how long living in the UK
  - follow any religion

- Main **daytime activity**
  - working or not: details

- **Other interests/activities**

- **Prostate cancer**
  - when diagnosed
  - treatment(s)

- **Overall health**
  - health conditions that **may affect diet/lifestyle**
    e.g. diabetes, heart problems, high blood pressure

- Importance and practicalities attached to **food and physical activity**
  - family/culture (who does the cooking/food purchase)
  - involvement in sports/food-related organisation (e.g. gym, community allotment)

2. Diet and lifestyle behaviours

- **Eating habits before diagnosis**
  - fruit and veg, dairy, meat, high fat/sugar
  - alcoholic drinks, juice
  - supplements, herbal products, others
- **Remained the same**?

IF NO

- **Describe changes**
  - adding/removing/change dietary pattern (overhaul)
- **When** change
- **Reasons** for change
  - perception of diet before diagnosis
  - beliefs (role of diet in prostate cancer)
  - social and/or family pressure

- **Reasons** for change *(continued)*
    - control
    - fear of recurrence/improve overall wellbeing
- **Maintenance**

- **Motivators and facilitators**
    - wife/partner/family (food purchase and cooking)
    - advice, information (advice from Health Professionals)
    - benefits to health/prostate cancer

- **Barriers (especially for maintaining changes)**
    - cost, time
    - risk to health/prostate cancer
    - treatment side-effects/fatigue/general health

- **Physical activity/exercise before diagnosis**
    - Types
    - how often, duration
    - where (gym, local leisure centre, parks)
- **Remained the same**?

IF NO

- Describe changes
- When change
- Reasons for change

NB: For PA, similar questions on motivators/facilitators, and barriers.

3. Information provision

- **Understanding and awareness**
    - explore what healthy eating means
    - know of any healthy eating/PA advice (source of advice)

- **Information about diet and lifestyle**
    - sources
    - views on information

- **Preferences**
    - what type of information
    - who should provide information (health professionals)
    - what format

4. Experience and views of dietary and lifestyle intervention

- **Participate in research study** - asked to change your **eating habits**

If YES

- where/how receive information
- types (delivery format)
- duration
- reasons for participating

***If NO,* why not? How to promote participation?**

- **Participate in research study** - asked to change your **physical activity**

If YES

- where/how receive information
- types (delivery format)
- duration
- reasons for participating

***If NO,* why not? How to promote participation?**

5. Preferences for intervention

- If we ask you to change your eating habits, for example cooked tomatoes, how would you feel about this? (soya products, fish, lycopene supplement)

If POSITIVE

- duration
- where/how receive information (leaflets, telephone)
- what would make it easier/harder
- when (after diagnosis/treatment)

***If NEGATIVE,* why? What would make it easier? Different type of intervention?**

- If we ask you to walk extra 30mins everyday, how would you feel about this?

If POSITIVE

- duration, type (brisk walking/stroll)
- where/how receive information (leaflets, telephone)
- what would make it easier/harder
- when (after diagnosis/treatment)

***If NEGATIVE,* why not? What would make it easier? Different type of intervention?**

Closing

- Thank you
- Any questions?
- Summary of results from the study?
- £15 voucher (thank you for your time)

*This topic guide was amended during data collection to include additional questions on concerns about body weight, familiarity with soya products and tomatoes as a feature of a traditional Caribbean diet. They were identified as salient topics in interviews with the first few participants.
