# Supplementary material for: Barriers and facilitators to healthy lifestyle and acceptability of a dietary and physical activity intervention among African Caribbean prostate cancer survivors in the UK: a qualitative study
Source: BMJ Open. 2017 Oct 15;7(10):e017217. doi: 10.1136/bmjopen-2017-017217 (PMC5652511; doi:10.1136/bmjopen-2017-017217)
Supplement: Supplementary file 1 [file bmjopen-2017-017217supp001.pdf]

Supplementary Material 2: Additional quotes from interviews

**Theme 1: Pre-cancer diet and lifestyle**	
Men who perceived themselves to have a healthy diet before prostate cancer did not change their diet	I don’t, I don’t make no changing. But as I says, I don’t overdo things…I normally look for things what is fat free…That’s why I, I eat, if I eat bread I eat brown bread. And if I drink tea with, tea with milk I will drink the green top. (Jamal)
The proposed dietary intervention-increasing tomato intake-was acceptable to men as they are commonly consumed as part of a traditional Caribbean diet	“Yeah, we cook with a lot of tomatoes. I cook it. That’s part of what we call the seasonal, you know, tomato, onions and thyme, garlic…So tomato is in everything and the seasoning. (Thomas)
Men who perceived themselves to be physically active before prostate cancer did not think they should be more active, and advocated moderation	… I don't run marathons, (Laugh) you know, I never did that, you know…maybe I could do a bit more [walking], but I don't. And at the moment I don't think I would, you know. Because I consider myself, I do keep fairly active. (Jonah)
	Well I, I exercise at the allotment (Laugh) as much as possible…So we’re active, you know, may- maybe not, no one is pushing you. What we, we do a little and I sit down, and don’t push yourself… (Colton)
Pre-existing injuries or health conditions as a barrier to physical activity	“…I used to do karate for 20 something years but, er, I have an injury so I give it up” (Albert).
	Just walking, yes. Because, like, I don’t want to do too much because of like, my asthma... (Shaun)
**Theme 2: Evidence: link between diet, lifestyle, and prostate cancer**	
Men did not think prostate cancer is linked to diet and lifestyle as they perceived themselves to have a healthy diet and lifestyle	I don't think it has anything to do with it. That's my view. I mean, you might differ. But I don't think it has anything to do with my diet. Because I think I eat all the right stuff. (Matthew)
Conflicting dietary messages	Er, I took cod liver capsule, I stopped because it’s no good after the time, they say they find out it’s no good for you, for, because, for long term they can give you prostate cancer too…Yeah. So if you listen, you know, [some of this diet] everything you do is no good, you know, so you eat nothing. (Albert)
Clinicians uncertain about the effectiveness of diet/lifestyle	he [doctor] can't give us any direction or any what to do at the moment, because they did say they’re not sure… So they are not sure yet, so obviously

on delaying or preventing prostate cancer progression	they can't give you any advice. All they are trying is to keep fit, you know, eat healthy and things like that. (Dennis)
**Theme 3: Coping with prostate cancer: Just get on with it**	
Men who wanted to return to a 'normal' life after prostate cancer were less likely to change or seek dietary/lifestyle information	Well really, to be honest, I never accept, I knew the idea of prostate leads to cancer, but I never thought that I had got it, I had it. I never think, "Oh I've got cancer." Nothing like that, no…but I was lucky enough I, I took it at the early stage, so it didn't have to develop… Just carry on with my normal life, you know. Do everything in moderation. (Fabian)
Social network and support as a facilitator to dietary and physical activity changes	…She's [sister] telling me that, you know, sugar and dairy products, that's the other things I must stop eating them things. (Laugh)...Since she told me that, I normally use skimmed milk to make my tea and I make porridge… I just try and do what she said. Less sugar, less dairy products. I just try to eat less of those things. I don't know. (Joseph)
	And it's just being in a room, with other people, that are trying to do something. There's a positive energy about being in a room… it's always better when you- well, when you walk in a pack, with people that are motivated the same way that you are. (Thomas)
**Theme 4: Ageing: Those were the days, this is me now**	
Ageing as a barrier to strenuous physical activity; adapting to changes to the body	Yeah I still do a lot of activities. Decorator, painting and decorating. I can do all that, but it's only now I, the ladder, my body is saying to me, "Don't climb the ladder much anymore."…suddenly you find the aches and pains and I can't climb the ladder anymore. (Fabian)
	Age. Cricket I am, I think last I played cricket was about maybe 15, 20 years ago and you feel as you get older you're restricted. I can't see myself running here and running there to catch a bus and that's not good for the heart. (Errol)
Ageing heightened men's awareness of their health, especially in regards to their body weight	… You know, it's a wise thing to change your diet, and I think I am reaching a certain age, you know… I think I should sacrifice and get rid of those things [starchy foods] and try and keep myself on a balanced diet where I could then chop my weight and look after myself… (Dennis)

	….I want to go to the gym soon…they reckon I'm putting on a bit too much weight so go and take off some of the weight (Laugh). (Sebastian)
**Theme 5: Autonomy: It has to be something I enjoy**	
Men who made changes out of necessity-chronic health conditions- broke their diet as they felt restricted	…but after you're diabetic you say okay, that's not good that's not good so you leave that out… Yes, so, so what I don't eating, I try to follow the rules as much as I can, but rules are meant to be broken. (Laugh). If we, if you obey all the rules all the time you miss half the fun. (Laugh) (Errol)
Preference in regards to incorporating tomatoes into diet	I eat tomatoes, but I don't like cooked ones…Fresh ones, I buy loads of them and I just squash them, cut them up and just eat them in my, eat them in bread and thing with my dinner. (Joseph)
Views on a dairy-free diet	Cut out the skimmed milk from, from my cereal, but I like my cereal, every morning. That would be difficult. I have my cereal every morning. I could cut out the cheese, but the milk. That's a difficult one for me. (Matthew)
Have to like the food to eat it, regardless of its benefits	I don't like, I can't eat the English type food most most 'cause a lot of things I don't I never eat it. They say it's good to eat, and I never eat it… (Joseph)
	…but it must be, not all fruit is palatable to me. I, I'm, I'm must like it to eat it, whether it's good for me or not I must like it. (Errol)
