# Supplementary material for: Barriers and facilitators to healthy lifestyle and acceptability of a dietary and physical activity intervention among African Caribbean prostate cancer survivors in the UK: a qualitative study
Source: BMJ Open. 2017 Oct 15;7(10):e017217. doi: 10.1136/bmjopen-2017-017217 (PMC5652511; doi:10.1136/bmjopen-2017-017217)
Supplement: Author's manuscript [file bmjopen-2017-017217.draft_revisions.pdf]

BMJ Open is committed to open peer review. As part of this commitment we make the peer review history of every article we publish publicly available.

When an article is published we post the peer reviewers' comments and the authors' responses online. We also post the versions of the paper that were used during peer review. These are the versions that the peer review comments apply to.

The versions of the paper that follow are the versions that were submitted during the peer review process. They are not the versions of record or the final published versions. They should not be cited or distributed as the published version of this manuscript.

BMJ Open is an open access journal and the full, final, typeset and author-corrected version of record of the manuscript is available on our site with no access controls, subscription charges or pay-per-view fees (http://bmjopen.bmj.com).

If you have any questions on BMJ Open's open peer review process please email editorial.bmjopen@bmj.com

BMJ Open

Barriers and facilitators to healthy lifestyle and acceptability of a dietary and physical activity intervention among African Caribbean prostate cancer survivors in the UK: a qualitative study

Journal:	*BMJ Open*
Manuscript ID	bmjopen-2017-017217
Article Type:	Research
Date Submitted by the Author:	07-Apr-2017
Complete List of Authors:	Er, Vanessa; University of Bristol School of Social and Community Medicine,<br>Lane, Athene; University of Bristol, School of Social and Community Medicine; NIHR Biomedical Research Unit in Nutrition, Diet and Lifestyle at University Hospitals Bristol NHS Foundation Trust and the University of Bristol<br>Martin, Richard; University of Bristol, Social Medicine; NIHR Biomedical Research Unit in Nutrition, Diet and Lifestyle at University Hospitals Bristol NHS Foundation Trust and the University of Bristol<br>Persad, Raj; Bristol Urological Institute, North Bristol NHS Trust, Southmead Hospital<br>Chinegwundoh, Frank; Barts Health NHS Trust, Department of Urology; City, University of London, School of Health Sciences<br>Njoku, Victoria; Barts Health NHS Trust, Department of Urology<br>Sutton, Eileen; University of Bristol School of Social and Community Medicine
**Primary Subject Heading**:	Qualitative research
Secondary Subject Heading:	Urology, Oncology, Nutrition and metabolism, Public health
Keywords:	Prostatic neoplasms, QUALITATIVE RESEARCH, diet, physical activity, Caribbean region, survivors

SCHOLARONE™
Manuscripts

Barriers and facilitators to healthy lifestyle and acceptability of a dietary and physical activity intervention among African Caribbean prostate cancer survivors in the UK: a qualitative study

Vanessa Er, J Athene Lane, Richard M Martin, Raj Persad, Frank Chinegwundoh, Victoria Njoku, Eileen Sutton

Corresponding author:

Dr Vanessa Er, School of Social and Community Medicine, University of Bristol, Canynge Hall, 39 Whatley Road, Clifton, Bristol, BS8 2PS, UK, E-mail: vanessa.er @bristol.ac.uk, Tel: +44 (0)117 928 7365.

Co-authors:

J Athene Lane, School of Social and Community Medicine, University of Bristol, 39 Whatley Road, Clifton, Bristol, BS8 2PS, UK. athene.lane@bristol.ac.uk

Richard M Martin, School of Social and Community Medicine, University of Bristol, 39 Whatley Road, Clifton, Bristol, BS8 2PS, UK. NIHR Bristol Nutrition Biomedical Research Unit. Level 3, University Hospitals Bristol Education & Research Centre, Upper Maudlin Street, Bristol, BS2 8AE, UK. MRC Integrative Epidemiology Unit, Oakfield House, Oakfield Grove, Bristol, BS8 2BN, UK. richard.martin@bristol.ac.uk

Raj Persad, Bristol Urological Institute, North Bristol NHS Trust, Southmead Hospital, Southmead Road, Bristol, BS10 5NB, UK. rajpersad@bristolurology.com

For peer review only

Frank Chinegwundoh, Department of Urology, Barts NHS Health Trust. The Royal London Hospital, Whitechapel Road, London E1 1BB, UK. School of Health Sciences, City, University of London. Northampton Square, London, EC1V 0HB, UK.

frank.chinegwundoh@nhs.net

Victoria Njoku, Department of Urology, Barts NHS Health Trust. The Royal London Hospital, Whitechapel Road, London, E1 1BB, UK. victoria.njoku@bartshealth.nhs.uk

Eileen Sutton, School of Social and Community Medicine, University of Bristol, 39 Whatley Road, Clifton, Bristol, BS8 2PS, UK. e.sutton@bristol.ac.uk

Keywords

Qualitative research, prostatic neoplasm, diet, physical activity, Caribbean region, survivors

Word Count

5023 words

Abstract

Objectives: Diet and lifestyle may have a role in delaying prostate cancer progression, but little is known about the health behaviours of Black British prostate cancer survivors despite this group having a higher prostate cancer mortality rate than their White counterparts. We explored the barriers and facilitators to dietary and lifestyle changes and the acceptability of a diet and physical activity intervention in African Caribbean prostate cancer survivors.

For peer review only

Design: We conducted semi-structured in-depth interviews and used thematic analysis to code and group the data.

Participants and setting: We recruited 14 African Caribbean prostate cancer survivors via letter or at oncology follow-up appointments using purposive and convenience sampling.

Results: A prostate cancer diagnosis did not trigger dietary and lifestyle changes in most men. This lack of change was underpinned by five themes: pre-cancer diet and lifestyle, evidence, coping with prostate cancer, ageing, and autonomy. Men perceived their diet and lifestyle to be healthy and were uncertain about the therapeutic benefits of these factors on prostate cancer recurrence. They considered a lifestyle intervention as unnecessary because their prostate-specific-antigen (PSA) level was kept under control by the treatments they had received. They believed dietary and lifestyle changes should be self-initiated and motivated, but were willing to make additional changes if they were perceived to be beneficial to health. Nonetheless, some men cited advice from health professionals and social support in coping with prostate cancer as facilitators to positive dietary and lifestyle changes. A prostate cancer diagnosis and ageing also heightened men's awareness of their health, particularly in regards to their body weight.

Conclusions: A dietary and physical activity intervention framed as helping men to regain fitness and aid post-treatment recovery aimed at men with elevated PSA may be appealing and acceptable to African Caribbean prostate cancer survivors.

For peer review only

Strengths and limitations

- Our study is one of the first to explore the dietary and lifestyle practices and information seeking behaviour of African Caribbean men after a prostate cancer diagnosis in the UK.

- Views and preferences for a dietary and physical activity intervention were sought from men from diverse sociodemographic backgrounds and with different prostate cancer clinical history.

- We struggled to recruit men on active monitoring, perhaps due to Black men's preference for having curative treatments rather than being monitored, or a tendency for their prostate cancers to be managed more aggressively by clinicians.

- We did not interview men's partners but it would be of interest to explore eating habits and food choices of the family with their partners as it may uncover discrepancies in men's narratives.

- This study reflects only intentions in response to a dietary and physical activity intervention which may differ from that in a real intervention.

For peer review only

Background

Prostate cancer is the second most common cancer in men worldwide, with over 1.1 million cases diagnosed in 2012.[1] In the UK, there are over 40,000 newly diagnosed cancer cases and 10,000 deaths each year.[2] Black British men are three times more likely to be diagnosed with prostate cancer than White men of the same age,[3] and have a 30% higher mortality rate

than White men in England.[4] Radical treatments for prostate cancer are often accompanied by adverse effects that negatively impact on men's quality of life,[5] and the prostate-specific antigen (PSA) test cannot distinguish the majority of men with indolent cancer from the minority with aggressive, fatal cancer.[6] Since diet and lifestyle are thought to play a pivotal role in prostate carcinogenesis,[7] attention has turned towards the development of dietary and lifestyle interventions to prevent the progression of prostate cancer.

The World Cancer Research Fund and the American Institute for Cancer Research (WCRF/AICR), which published the most comprehensive and authoritative report on environmental exposures and cancer, concluded that there is some evidence that selenium and foods rich in lycopene probably decrease the risk of prostate cancer while diets high in calcium increases prostate cancer risk.[7] The WCRF/AICR continuous update project report published in 2014 found strong evidence for a link between body fatness and increased risk of advanced prostate cancer.[8] Physical activity has been linked to a lower risk[9,10] of prostate cancer progression and mortality, and has also been shown to alleviate the adverse effects of prostate cancer treatment.[11]

However, current evidence suggests that prostate cancer survivors have sub-optimal diet and lifestyle profiles[12-15] and the evidence-base to support the development of successful and conclusive interventions is lacking and of poor quality.[16] There is a high prevalence of obesity among prostate cancer survivors,[12,14,15] and the proportion of prostate cancer survivors consuming '5 a day' of fruits and vegetables in most studies was relatively low, ranging from 15.6% to 30.7%.[12,13,15] Similarly, it has been reported that over 50% of prostate cancer survivors are inactive.[12-14] Whilst there is a body of literature on the health behaviours of prostate cancer survivors, most studies were conducted in predominantly White

For peer review only

population and in the US, with limited qualitative studies exploring the facilitators of, and barriers to dietary and lifestyle changes.[17-21]

To our knowledge, there is no qualitative study on the health behaviours of African Caribbean men after a prostate cancer diagnosis. Although Black men in general are at higher risk of prostate cancer, the African Caribbean community in the UK is more established, with a larger elderly population[22] and hence more likely to be affected by prostate cancer. It is important to understand the health behaviours and needs of African Caribbean prostate cancer survivors, so as to inform the development of a dietary and lifestyle intervention which is both acceptable to this population and effective in improving prostate cancer outcomes. The aim of this qualitative study was to explore the facilitators and barriers to dietary and lifestyle changes, and the acceptability of a dietary and physical activity intervention among African Caribbean prostate cancer survivors to inform the development of a high-quality diet and physical activity randomised controlled trial.

Methods

Study population and ethical approval

We recruited men aged 18 and above who self-identified as African Caribbean, and had a clinically–confirmed prostate cancer diagnosis from two large cities in the UK by invitation letter through their consultants and in-person at oncology follow-up clinics. We used purposive sampling to select men across a range of ages, socioeconomic positions, marital status and treatment types to obtain a representative sample.[23] These factors have been identified in the literature to influence dietary and lifestyle changes after a prostate cancer diagnosis.[24] As a result of slow recruitment at the first site and time constraints – this study was conducted as part of a doctoral degree - we used convenience sampling[23] and focused our

For peer review only

recruitment on another site with larger African Caribbean community. Ethical approval was provided by the Greater Manchester West Research Ethics Committee (13/NW/0878).

In-depth interviews

All participants provided their written informed consent to take part in the study, for their interviews to be audio-recorded and quotes to be published. A researcher (VE) carried out in-depth, semi-structured interviews with participants at their homes in 2015. Interviews lasted between 45 minutes and 1 hour 40 minutes and participants received a £15 supermarket voucher at the end of the interview as a thank you for taking part. VE used an interview topic guide to direct discussions on men's dietary and lifestyle practices before and after a prostate cancer diagnosis, the reasons for dietary and/or lifestyle changes (or lack of), sources and views on dietary and lifestyle information, and views on a proposed dietary and physical activity intervention for prostate cancer survivors, which consists of increasing tomato intake, a dairy-free diet and 30 minute of brisk walking a day. The topic guide was informed by the literature, reviewed by a public and patient involvement group, and was refined twice based on preliminary findings from early interviews to include topics on body weight, familiarity with soya products, and tomatoes being a feature in the Caribbean diet.

Data analysis

The interviews and data analysis were carried out concurrently.[25] This was an iterative process such that salient issues or topics raised in early interviews could be explored or follow-up in subsequent interviews. All interviews were anonymised, transcribed verbatim and analysed using thematic analysis[26] with the aid of a qualitative data analysis computer software package (NVivo 10). The researcher (VE) used an inductive approach to code the data in 'chunks' to keep the context where the data originated, and developed an initial

For peer review only

coding frame.[26] A portion of transcripts was coded independently by another researcher (ES) to ensure coding was consistent and was a balanced representation of participants' views. Throughout the coding process, extracts of data across the dataset were compared to check for similarities and differences.[26] The coding framework was refined and revised by VE and ES as the analysis progressed. We identified, grouped and conceptualised patterns within and across the dataset which are salient to the participants into themes.[26] VE and ES discussed any discrepancies and finalised the themes. All names presented here refer to pseudonyms.

Results

The characteristics of the participants are presented in **Table 1**.

Table 1 Characteristics of participants

Pseudonym	Age	Marital status	Years since diagnosis	Cancer stage	Treatment
Aaron	79	Married/Partner	≤1	Localised	Not started[a]
Dennis	56	Married/Partner	≤1	Localised	Not started[a]
Jonah	75	Married/Partner	>10	Locally advanced	RT/HT
Fabian	73	Married/Partner	>1 and <5	Locally advanced	RT/HT
Colton	77	Married/Partner	>10	Locally advanced	RT/HT
Albert	65	Married/Partner	≥5 & <10	Localised	RT/HT
Jamal	70	Single/Widowed	>1 and <5	Locally advanced	RT/HT
Matthew	80	Single/Widowed	>10	Locally advanced	RT/HT
Irvin	79	Single/Widowed	>1 and <5	Localised	RT/HT
Errol	76	Single/Widowed	≤1	Locally advanced	RT/HT
Shaun	68	Married/Partner	≤1	Localised	RT/HT
Joseph	63	Married/Partner	≤1	Localised	Prostatectomy
Sebastian	61	Married/Partner	≤1	Localised	Prostatectomy
Thomas	52	Married/Partner	≤1	Localised	Prostatectomy

RT/HT: radiotherapy and adjuvant hormone therapy.
[a] Had not started treatment at the time of interview.

Five themes were identified from the analysis which captured the barriers and facilitators to dietary and lifestyle changes, the rationale for seeking (or not) dietary and lifestyle information, and men's views on the target population, timing, and acceptability of a proposed dietary and physical activity intervention. Additional quotes relating to each theme are provided in **Supplementary Material 1.**

Theme 1: Pre-cancer diet and lifestyle

Most men who perceived their diet to be healthy before a prostate cancer diagnosis ate as usual. Some men had already made positive dietary changes prompted by other health problems such as diabetes, while others questioned what changes could be made if they were already eating healthily.

> *Well, but erm - change to what? (Laugh) What do you change to, in those circumstances? If you eat, you know, if you eat healthy food, meat, vegetables, salads, fruits, what do you change to? (Jonah)*

Thus, most of the men did not seek further dietary information after a prostate cancer diagnosis as they perceived themselves as having a healthy lifestyle and knowing what constitutes a healthy diet. When men were asked for their views on a dietary intervention which would require them to eat more tomatoes and cut out dairy products, they were amenable to it because it complemented their existing diet. They explained that tomatoes are commonly consumed as part of a traditional Caribbean diet, while dairy products only account for a small fraction of it. Similarly, a proposed physical activity intervention which involves brisk walking for 30 minutes a day was considered achievable, with several men already doing it in their daily lives.

For peer review only

Men who perceived themselves to be active before prostate cancer also did not think they needed to increase their physical activity. For men, being active meant being up on their feet and 'doing things', but they advocated moderation. As one participant (Errol) noted: *"I do the exercise in my work. 'Cause when you're a builder, you don't sit, you don't do nothing, you have to move around all the time, you know. So that to me enough exercise."* A few men wanted to be more active but they were impeded by health conditions or sports injuries. Conversely, men who made changes acknowledged that their diet and lifestyle before prostate cancer could have been better and a prostate cancer diagnosis acted as a catalyst for the change.

> *…But since I was diagnosed, er, we just, I would say about, the beef probably come out, we cut down beef a bit, you know? … they always say too much red beef is not good for you and that. So we just cut it down, you know? (Albert)*

Theme 2: Evidence: link between diet, lifestyle, and prostate cancer

Men's views on the causes of cancer varied, ranging from genetic factors, fate or chance, stress (not enough rest), diet and lifestyle to food production (chemicals). However, the majority of men were aware that Black men have higher risk of prostate cancer, and some described it as an 'old man's complaint'. A few men cited a lack of evidence that prostate cancer is linked to diet and lifestyle to support their view. This was contrasted with men's belief that smoking increases the risk of cancer due to widely established evidence that it causes lung cancer.

> *…up until now they haven't come out with err, err, anything positive to say, "This is the result, this is the main reason why black men from the Caribbean or West Africa*

For peer review only

have a, how I say, the most higher rate."…So I don't think that I am in any position to think of, because of our diet or because of what we eat, do you understand? (Dennis)

Men who believed that prostate cancer is not connected to diet were less likely to seek dietary information. This was partly due to their mistrust of the dietary messages from media which they regarded as conflicting. They preferred receiving information from health professionals who they regarded as experts and a trusted source of health information, and that had a positive influence on their health behaviour. For example, one man (Colton) started eating tomatoes and drinking pomegranate juice because his clinicians informed him that it could be beneficial for prostate cancer: *"she [dietician] said with, with the prostate cancer that I've got I should eat a lot of tomato. Tomato is good for the prostate cancer. My doctor before, Doctor M, told me that if I drink pomegranate juice it's a little bit helpful as well…That's what I buy, we buy, things, we buy pomegranate juice."* However, only a few men reported that they received dietary or lifestyle advice from health professionals, with some explaining that their doctor was uncertain about the effectiveness of diet and lifestyle on prostate cancer.

There were however a small number of men who believed that diet and lifestyle is important for cancer prevention, but did not think it has any impact on cancer progression, particularly if their treatment was effective: *"So, so what the difference that it make to, it's not going to affect me now because I'm, I've [passed that stage] with the prostate cancer" (Albert).* Consequently, men suggested that a dietary and physical activity intervention should be offered before treatment, as the effect of the intervention would not be confounded by the treatment, and if the intervention works, treatment would not be necessary. The significance of PSA became apparent when men explained their views, as a high or rising PSA level after treatment was perceived as indicative of recurrent prostate cancer. Accordingly, they

For peer review only

believed that interventions should be targeted at men who have a high PSA level and were more likely to adopt dietary or lifestyle advice if it was shown to reduce or keep PSA level low.

Theme 3: Coping with prostate cancer: just get on with it

Men had differing approaches to coping with prostate cancer which influenced whether they made positive changes to their diet and lifestyle. Those who were sanguine about prostate cancer tended to 'take it as it comes' and thus less likely to change their diet and lifestyle and were not interested in gaining further dietary and lifestyle information. Similarly, men who considered their cancer as treated just wanted to move on and not dwell upon it further.

> *Well me don't really want to get no more information, because so far I'm not living with the thing again. So I don't really want to get no more information, I just try to do, the, my best, try to help myself in many ways. (Jamal)*

These approaches were directly contrasted with those of men who were actively seeking ways to alleviate the side effects they experienced and wanting to be fit again. Thomas spoke about exercise as a way of making himself feel better as it felt like he was doing something for his body, rather than relying on medication for depression or pain: "*If there's something that you need, to motivate yourself and exercise, might trigger it off. I'm not sure, but I know that sitting down, taking tablets, isn't going to do it, or not even diet, to that point."*

It became evident that social networks and support were important for men in coping with prostate cancer, and facilitated positive dietary and lifestyle changes. Partners, family and friends acted as a 'change agent' to enable these changes. The role of partner or wife in

For peer review only

initiating dietary changes and encouraging men to live healthier is highlighted in this comment.

> *… more of less it's the wife that is leading the process [dietary change] really. (Laugh) Yeah, she's the one doing the shopping, she's the one providing the food. And now she's, she's going and, and do all the shopping and buy whatever she thinks that's good for our health. (Dennis)*

Several men commented on the importance of social support to stay physically active. They were very positive about group exercise citing shared purpose and motivation as an attraction to them, as one participant (Matthew) commented: *"what group exercises do is to encourage you and encourage others to follow. That is why I used to like going to the gym, 'cause you had people there who were there for the same purpose."*

Whether men made dietary and lifestyle changes was also dependent on their priorities and concerns after cancer treatment. Men typically experienced side effects from treatments including incontinence and erectile dysfunction. Thus, coping and adapting to these side effects were their main priorities. Primarily, incontinence was reported as a barrier to physical activity.

> *I want to go to the gym soon, but like I said, because I'm so wet I won't have – I'm hoping that it will ease up a little bit so I can get to do something else….(Sebastian)*

Loss of income because of a prostate cancer diagnosis could also have a knock-on effect on opportunities for physical activity. Sebastian who was self-employed had financial worries and his main priority was to return to work, for without an income, he could not afford to pay for gym membership.

For peer review only

Theme 4: Ageing: those were the days, this is me now

Most men used to play sports and acknowledged the health benefits of physical activity, but old age was the most commonly cited reason for not doing strenuous physical activity. For men who had retired, life after work was portrayed as a time to rest (slow down) and for leisure. Most men (aged 70 and above) viewed themselves as too old to be playing sports and gentle exercise as safer and more appropriate for their age. Therefore, a brisk walking intervention was perceived as safe and acceptable by men in the study.

> *I'm too old to go to the gym man. I don't want to go to the gym to get a heart attack. (Laughter) Because I don't know, I don't go there [to lift up] these big great things, I go…on the treadmill. (Laugh) I used to go on it but I don't want to do all these type of things now. (Jamal)*

Men made frequent references to their body when describing their physical limitations. They also talked about having to accept and adapt to the changes in their body as they aged. As one participant (Joseph) noted: *"Age is against me now (laugh). I don't feel much pain, but you can tell on the body. Your body can't cope with that. I know it can't cope with it anymore."*

Younger men (under 65 years old) however had no qualms about performing higher intensity physical activity. For Thomas, being back in the gym and having the ability to do the exercises just as before, was a sign of recovery - "*erm- because I've been in a gym before, it's going back to an old friend, and actually familiarising yourself with it, and being told- and telling yourself that you- you're back on the road to recovery…To be back in that gym environment again, it- it was lovely…*" Similarly, Dennis who had a prostate biopsy before the interview hoped to continue playing cricket for his club - *"Well, I used to play every*

For peer review only - http://bmjopen.bmj.com/site/about/guidelines.xhtml

Sunday…but since I had the operation I haven't played…when I am feeling healthy and okay I will".

On the other hand, men had more awareness of their health as they aged because they perceived that they were more susceptible to illness, so having a healthy diet and lifestyle became more relevant to them. For the men in this study, ageing also shifted the focus from providing for the family to taking care of themselves. There was also a perception espoused by men that getting older made one wiser and thus more sensible. Some men noticed that they put on weight more easily as they aged, mainly because they were less active than before. That prompted men to eat less, cut down food portion sizes, alcohol intake or exercise more.

> *Yeah, that's why I said don't want to eat fatty things. I don't want to be bloated out, put it that way…Yeah, yeah, I don't want to get big, fat and clumsy man, where I can't move about and things like that. So I, I watch, I know what I'm eating. (Jamal)*

The younger men who experienced side effects from prostate cancer treatments, especially incontinence and fatigue, and thus unable to perform strenuous physical activity also expressed concerns about their weight and wanted to regain fitness to cope with these side effects.

> *… she [trainer] said, "You've got a bigger belly than you had before, and if you want- what do you want to get out of this?" And I said, "I'd like to probably get my shape back, and just lose a little bit of weight…" (Thomas)*

For peer review only - http://bmjopen.bmj.com/site/about/guidelines.xhtml

Theme 5: Autonomy: it has to be something I enjoy

Overall, men believed that an individual has to be personally motivated to make dietary and lifestyle changes so that they would be maintained long-term. Men who made changes to their diet and lifestyle out of necessity due to co-morbidities such as diabetes, felt restricted and thus sometimes broke the rules. Although the proposed dietary intervention which required men to increase intake of tomatoes was acceptable for most men, they expressed different preferences in regards to taste and ways of incorporating tomatoes in their diet. A few men favoured whole tomatoes over lycopene supplement (tablets) because they were suspicious of its content and production process.

> *It would be easier, but- but, I mean, it's- yeah, it would be easier, without a doubt. If you could take a tomato tablet that does it, but then it's what goes into making the tablet. With a tomato, a fresh tomato, you know what you're getting, don't you? (Thomas)*

Similarly, men in general were amenable to a dairy-free diet but they had reservations about substituting dairy products with soya, because they were unfamiliar with soya products and perceived dairy substitutes as unnecessary due to a low dairy intake. Nevertheless, there were a few men who were hesitant to give up milk which they usually consumed with breakfast cereals or in porridge. Some men also commented that they have to enjoy or like the food to eat it, regardless of its health benefits. One man rejected the idea of eating tomatoes every day despite being told of the potential protective effect of tomatoes from prostate cancer – *"No I won't eat all that…normally I use one for the salad - if I make salad – [I wouldn't want to do]… You know? I don't eat every day…I don't feel like doing it, and I don't want to do it. (Laugh)"* (Irvin).

For peer review only

Finally, the majority of the men interviewed preferred to walk on their own instead of taking part in a group exercise, even though they recognised the support and motivation that it could provide. Men explained that each person has his own schedule and routine, thus it is easier to walk on their own and at a pace that suited them.

> *Na, because again I have to, they want, when I'm ready to do it they won't be ready, that's the problem. That's why I have to do my own. (Albert)*

Discussion

Most men in our study did not change their diet and lifestyle after a prostate cancer diagnosis. The lack of change was underpinned by five key themes, as described previously. The implications for research and practice are described below.

Design of dietary and lifestyle intervention for prostate cancer survivors

The proposed dietary and physical activity intervention was acceptable to men in this study, but they suggested it should be aimed at men with an elevated or rising PSA level, including those who are on active monitoring. The majority of dietary and lifestyle interventions for cancer survivors have been conducted after treatment with the aim of improving general health, and quality of life, but this finding suggests that men may be more interested in the clinical effect of interventions. Consequently, men perceived the proposed intervention as unnecessary because their PSA level was kept under control by the treatments they had received, which supports the findings of a previous study[24] and highlights the significance of using PSA as an outcome measure, despite criticism of its usefulness as a surrogate end-point in prostate cancer trials.[27,28] It also illustrates the challenges of informing and convincing men that dietary or lifestyle factors may act on prostate cancer independently of PSA.

For peer review only

This study also showed that men valued autonomy in their food and lifestyle choices and they might change their diet and lifestyle if it was deemed necessary and beneficial for health. Similar studies examining healthy eating using gender theory found that resistance among men was linked to masculine ideals of autonomy and rationality.[29] The authors suggest that healthy eating initiatives and materials could be tailored to men by emphasising personal choices and responsibility.[29] Most men in our study preferred individual over group exercise, which differed from previous studies reporting that men enjoyed the opportunity to socialise when exercising.[21,30] Nonetheless, men were willing to participate in weekly group exercise provided that a time and place that is convenient for all could be found. Therefore, either a choice of home-based intervention only or in combination with weekly supervised group exercise may appeal to African Caribbean prostate cancer survivors.

Provision of dietary and lifestyle advice and support

Evidence from existing literature suggests that most men were uncertain about the role of diet and lifestyle in preventing prostate cancer progression or recurrence, but a strong belief in cancer-diet relationship was a predictor of dietary changes.[17-19,24,31] As with previous studies,[17,20,21,24,31] our findings suggest that advice from health professionals can strengthen men's beliefs on the relationship of prostate cancer with diet and lifestyle, influencing men to make positive lifestyle changes. Most men in our study did not recall receiving dietary or lifestyle information from health professionals, perhaps due to their hesitance on providing dietary and lifestyle advice because of a lack of evidence on diet and prostate cancer and awareness of relevant guidelines for cancer survivors.[24,32] Nonetheless, it is becoming clear that obesity is associated with higher risk of advanced and fatal prostate cancer,[8] and health professionals could potentially play a role in influencing men's health behaviour.

For peer review only

Awareness of supporting services was low among our participants. The UK National Cancer Survivorship Initiative (NCSI) recommend a 'recovery package' which consists of a combination of interventions, including a patient education and support event (health and wellbeing clinic) to be offered to all cancer survivors.[33] A report by the London Cancer Alliance found that there was no standardised definition or structure for health and wellbeing clinics in their partner NHS trust providers.[34] Therefore, more research is needed in designing and evaluating the referral pathways for healthy lifestyle services, and developing a coherent model for health and wellbeing clinics.

Active ageing and body weight

In line with previous research,[35,36] misconceptions about the harms or risks of strenuous physical activity in old age such as heart attack and feeling breathless were shared by men in this study. Older men also felt that going to the gym was incompatible with their age as for them it was typically associated with young people 'pumping iron'. Although there has been much effort in the UK and developed countries to promote active ageing, this shows that further work is needed to challenge prevailing social norms on ageing and physical activity to ensure men stay active as they age. Older African Caribbean prostate cancer survivors may need to be persuaded that resistance exercise is beneficial to their health especially for preventing loss of muscle mass in men on androgen deprivation therapy.

Ageing or being diagnosed with prostate cancer also heightened men's awareness of their health and prompted several men to make positive changes to their diet and lifestyle for maintenance of general health. Concerns about body weight among older men and prostate cancer survivors have been reported in several studies.[18,24,29] In this study, men wanted to maintain a healthy weight to avoid having a 'big belly'. This differs from previous studies

For peer review only

which found that men lose weight for health purposes rather than for appearance (body image).[37,38] Men in this study watched their weight by eating smaller portion sizes, having light meals (less starchy food) and increasing their physical activity, contradicting previous studies which found that men preferred to lose weight through exercising because it is regarded as a masculine pursuit,[37] while 'dieting' practices such as increasing fruits and vegetable intake and reducing food portion sizes have been perceived to be feminine.[38,39]

Younger men also expressed interest in losing weight and regaining fitness after a period of inactivity due to side-effects of treatment. Similar to previous studies, men who experienced urinary incontinence were unsure about the type and intensity of physical activity that they could perform for fear of exacerbating their condition.[20,21,30] Thus, a supervised physical activity intervention could be sold to men as 'killing two birds with one stone', helping them to lose weight and alleviate the side effects of treatments. A physical activity intervention could also be framed as a way for men to take charge of their recovery for better health.

Strengths and limitations

Our study is one of the first to explore the dietary and lifestyle practices and information seeking behaviour of African Caribbean men after a prostate cancer diagnosis in the UK. Additionally, views and preferences for a dietary and physical activity intervention were sought from men to inform the design of a tailored intervention. It included men from diverse sociodemographic backgrounds and with different prostate cancer clinical history.

We struggled to recruit men on active monitoring, perhaps due to Black men's preference for having curative treatments rather than being monitored, or a tendency for their prostate cancers to be managed more aggressively by clinicians.[40] We did not interview men's

For peer review only

partners. It would be of interest to explore eating habits and food choices of the family with their partners as it may uncover discrepancies in men's narratives. There was an indication that men preferred to consume tomatoes over a lycopene supplement, which requires further investigation. Furthermore, questions on format of delivery (telephone-based, print-based, supervised physical activity) and ways of monitoring adherence (food diary, pedometers) were not explored in all interviews, precluding comparisons between men. In future research, clear and simple visual aids could be used to present and facilitate discussions on the proposed intervention. Finally, this study reflects only intentions in response to a dietary and physical activity intervention; this may differ from that in a real intervention.

Conclusions

A prostate cancer diagnosis did not trigger dietary and lifestyle changes in this group of men who mostly perceived their diet and lifestyle to be healthy and were uncertain about the therapeutic benefits of diet and lifestyle on prostate cancer recurrence. They considered an intervention as unnecessary because their PSA level was kept under control by the treatments they had received. Men believed dietary and lifestyle changes should be self-initiated and motivated but were willing to make additional changes if they were perceived to be beneficial to health. Nonetheless, some men cited advice from health professionals and social support in coping with prostate cancer as facilitators to positive dietary and lifestyle changes. Furthermore, a prostate cancer diagnosis and ageing heightened men's awareness of their health, particularly in regards to their body weight. Therefore, a dietary and physical activity intervention which enhances men's autonomy, framed as helping men to regain fitness and aid post-treatment recovery, and is aimed at men with elevated PSA may be appealing and acceptable to African Caribbean prostate cancer survivors.

For peer review only

Declarations

Contributing statement

VE, ES, JAL, and RMM conceived the study; VE, ES, FC, RJ and VN contributed to the design and conduct of the research, and acquisition of data; VE and ES analysed the data; VE, ES, RMM and JAL wrote the first draft of the paper; ES, JAL, and RMM provided supervision; VE, ES, JAL, and RMM have primarily responsibility for final content. All authors were involved in revising the paper, and read and approved the final version of the paper.

Acknowledgement

The authors would like to thank all participants for sharing their views and experience; Dr. Mona Jeffreys and the Bristol Nutrition BRU prostate patient and public involvement group for their input into the study design and documents; and all staff who had helped with the recruitment of participants, especially Dr. Karen Tipples.

Disclaimers

The views expressed herein are those of the authors and do not necessarily reflect those of Cancer Research UK, the NHS, the NIHR, or the Department of Health.

Competing interest

We have read and understood BMJ policy on declaration of interest and declare that we have no competing interests.

For peer review only

Funding

This work was supported by the National Institute for Health Research (NIHR) Bristol Nutrition Biomedical Research Unit. The unit is a partnership between University Hospitals Bristol NHS Foundation Trust and the University of Bristol. VE was a recipient of a 3-year PhD studentship at the Bristol Nutrition Biomedical Research Unit from 2012-2015. RMM and JAL are recipients of CRUK programme funding (C18281/A19169).

Data sharing statement

The datasets generated and/or analysed during the current study are not publicly available as participants may be identifiable, but are available from the corresponding author on reasonable request.

Ethics approval and consent to participate

Ethical approval was provided by the Greater Manchester West Research Ethics Committee (13/NW/0878). All participants provided their written informed consent to take part in the study.

References

1. Ferlay J, Soerjomataram I, Dikshit R, et al. Cancer incidence and mortality worldwide: sources, methods and major patterns in GLOBOCAN 2012. *Int J Cancer* 2015;136(5):E359-86.
2. Office for National Statistics. Cancer incidence and mortality in the United Kingdom 2008-2010 http://www.ons.gov.uk/ons/index.html2012 [accessed September 2013.
3. Ben-Shlomo Y, Evans S, Ibrahim F, et al. The risk of prostate cancer amongst black men in the United Kingdom: The PROCESS cohort study. *Eur Urol* 2008;53(1):99-105.
4. Hounsome L, Verne J. Mortality from prostate cancer. UK: National Cancer Intelligence Network, 2012.
5. Bangma CH, Roemeling S, Schroder FH. Overdiagnosis and overtreatment of early detected prostate cancer. *World J Urol* 2007;25(1):3-9.

For peer review only

6. Lilja H, Ulmert D, Vickers AJ. Prostate-specific antigen and prostate cancer: prediction, detection and monitoring. *Nat Rev Cancer* 2008;8(4):268-78.
7. World Cancer Research Fund, American Institute for Cancer Research. Food, nutrition, physical activity, and the prevention of cancer: a global perspective. Washington DC: AICR 2007.
8. World Cancer Research Fund International, American Institute for Cancer Research. Diet, nutrition, physical activity and prostate cancer. Continuous update project: World Cancer Research Fund, 2014.
9. Kenfield SA, Stampfer MJ, Giovannucci E, et al. Physical activity and survival after prostate cancer diagnosis in the health professionals follow-up study. *J Clin Oncol* 2011;29(6):726-32.
10. Bonn SE, Sjolander A, Lagerros YT, et al. Physical activity and survival among men diagnosed with prostate cancer. *Cancer Epidemiol Biomarkers Prev* 2015;24(1):57-64.
11. Gardner JR, Livingston PM, Fraser SF. Effects of exercise on treatment-related adverse effects for patients with prostate cancer receiving androgen-deprivation therapy: a systematic review. *J Clin Oncol* 2014;32(4):335-46.
12. LeMasters TJ, Madhavan SS, Sambamoorthi U, et al. Health behaviors among breast, prostate, and colorectal cancer survivors: a US population-based case-control study, with comparisons by cancer type and gender. *J Cancer Surviv* 2014;8(3):336-48.
13. Blanchard CM, Courneya KS, Stein K. Cancer survivors' adherence to lifestyle behavior recommendations and associations with health-related quality of life: Results from the American Cancer Society's SCS-II. *J Clin Oncol* 2008;26(13):2198-204.
14. Coups EJ, Ostroff JS. A population-based estimate of the prevalence of behavioral risk factors among adult cancer survivors and noncancer controls. *Prev Med* 2005;40(6):702-11.
15. Rogers LQ, Courneya KS, Paragi-Gururaja R, et al. Lifestyle behaviors, obesity, and perceived health among men with and without a diagnosis of prostate cancer: A population-based, cross-sectional study. *BMC Public Health* 2008;8:43-58.
16. Hackshaw-McGeagh LE, Perry RE, Leach VA, et al. A systematic review of dietary, nutritional, and physical activity interventions for the prevention of prostate cancer progression and mortality. *Cancer Causes Control* 2015;26(11):1521-50.
17. Satia JA, Walsh JF, Pruthi RS. Health behavior changes in White and African American prostate cancer survivors. *Cancer Nurs* 2009;32(2):107-17.
18. Coa KI, Smith KC, Klassen AC, et al. Exploring Important Influences on the Healthfulness of Prostate Cancer Survivors' Diets. *Qual Health Res* 2015;25(6):857-70
19. Mroz LW, Chapman GE, Oliffe JL, et al. Prostate cancer, masculinity and food. Rationales for perceived diet change. *Appetite* 2010;55(3):398-406.
20. Craike MJ, Livingston PM, Botti M. An exploratory study of the factors that influence physical activity for prostate cancer survivors. *Support Care Cancer* 2011;19(7):1019-28.
21. Keogh JW, Patel A, MacLeod RD, et al. Perceived barriers and facilitators to physical activity in men with prostate cancer: possible influence of androgen deprivation therapy. *Eur J Cancer Care (Engl)* 2014;23(2):263-73.
22. Centre for Policy on Ageing (UK). The ageing of the ethnic minority populations of England and Wales: findings from the 2011 census. UK, 2013.
23. Ritchie J, Lewis J, Nicholls CM, et al. Qualitative research practice: A guide for social science students and researchers: Sage 2013.

For peer review only

24. Avery KN, Donovan JL, Horwood J, et al. The importance of dietary change for men diagnosed with and at risk of prostate cancer: a multi-centre interview study with men, their partners and health professionals. *BMC Fam Pract* 2014;15:81.
25. Glaser B, Strauss, A. The discovery of grounded theory: strategies for qualitative research. New York: Aldine 1967.
26. Braun V, Clarke V. Using thematic analysis in psychology. *Qualitative Research in Psychology* 2006;3(2):77-101.
27. Thompson IM. PSA: a biomarker for disease. A biomarker for clinical trials. How useful is it? *J Nutr* 2006;136(10):2704S.
28. Collette L. Prostate-specific antigen (PSA) as a surrogate end point for survival in prostate cancer clinical trials. *Eur Urol* 2008;53(1):6-9.
29. Gough B, Conner MT. Barriers to healthy eating amongst men: a qualitative analysis. *Soc Sci Med* 2006;62(2):387-95.
30. Bourke L, Doll H, Crank H, et al. Lifestyle Intervention in Men with Advanced Prostate Cancer Receiving Androgen Suppression Therapy: A Feasibility Study. *Cancer Epidemiology Biomarkers & Prevention* 2011;20(4):647-57.
31. Horwood JP, Avery KN, Metcalfe C, et al. Men's knowledge and attitudes towards dietary prevention of a prostate cancer diagnosis: a qualitative study. *BMC Cancer* 2014;14:812.
32. Sutton E, Hackshaw-McGeagh LE, Aning J, et al. The provision of dietary and physical activity advice for men diagnosed with prostate cancer: a qualitative study of the experiences and views of health care professionals, patients and partners. *Cancer Causes Control* 2017;28(4):319-29.
33. Department of Health (England), Macmillan Cancer Support, NHS Improvement (England). Living with & beyond cancer: taking action to improve outcomes London, UK, 2013.
34. London Cancer Alliance. Health and wellbeing events mapping. UK, 2015.
35. Grant BC. 'You're never too old': beliefs about physical activity and playing sport in later life. *Ageing and Society* 2001;21:777-98.
36. Cousins SOB. Exercise, aging, and health: Overcoming barriers to an active old age: Taylor & Francis 1998.
37. De Souza P, Ciclitira KE. Men and dieting: A qualitative analysis. *J Health Psychol* 2005;10(6):793-804.
38. Gough B. 'Real men don't diet': an analysis of contemporary newspaper representations of men, food and health. *Soc Sci Med* 2007;64(2):326-37.
39. Jensen KO, Holm L. Preferences, quantities and concerns: socio-cultural perspectives on the gendered consumption of foods. *Eur J Clin Nutr* 1999;53(5):351-59.
40. Hounsome L, Iles M, Verne J. Treatment routes in prostate cancer. UK: The National Cancer Intelligence Network, 2012.

For peer review only

Supplementary Material 1: Additional quotes from interviews

**Theme 1: Pre-cancer diet and lifestyle**	
Men who perceived themselves to have a healthy diet before prostate cancer did not change their diet	I don't, I don't make no changing. But as I says, I don't overdo things…I normally look for things what is fat free…That's why I, I eat, if I eat bread I eat brown bread. And if I drink tea with, tea with milk I will drink the green top. (Jamal)
The proposed dietary intervention-increasing tomato intake-was acceptable to men as they are commonly consumed as part of a traditional Caribbean diet	"Yeah, we cook with a lot of tomatoes. I cook it. That's part of what we call the seasonal, you know, tomato, onions and thyme, garlic…So tomato is in everything and the seasoning. (Thomas)
Men who perceived themselves to be physically active before prostate cancer did not think they should be more active, and advocated moderation	… I don't run marathons, (Laugh) you know, I never did that, you know…maybe I could do a bit more [walking], but I don't. And at the moment I don't think I would, you know. Because I consider myself, I do keep fairly active. (Jonah)
	Well I, I exercise at the allotment (Laugh) as much as possible…So we're active, you know, may- maybe not, no one is pushing you. What we, we do a little and I sit down, and don't push yourself… (Colton)
Pre-existing injuries or health conditions as a barrier to physical activity	"…I used to do karate for 20 something years but, er, I have an injury so I give it up" (Albert).
	Just walking, yes. Because, like, I don't want to do too much because of like, my asthma... (Shaun)
**Theme 2: Evidence: link between diet, lifestyle, and prostate cancer**	
Men did not think prostate cancer is linked to diet and lifestyle as they perceived themselves to have a healthy diet and lifestyle	I don't think it has anything to do with it. That's my view. I mean, you might differ. But I don't think it has anything to do with my diet. Because I think I eat all the right stuff. (Matthew)
Conflicting dietary messages	Er, I took cod liver capsule, I stopped because it's no good after the time, they say they find out it's no good for you, for, because, for long term they can give you prostate cancer too…Yeah. So if you listen, you know, [some of this diet] everything you do is no good, you know, so you eat nothing. (Albert)
Clinicians uncertain about the effectiveness of diet/lifestyle on delaying or preventing	he [doctor] can't give us any direction or any what to do at the moment, because they did say they're not sure… So they are not sure yet, so obviously

For peer review only

prostate cancer progression	they can't give you any advice. All they are trying is to keep fit, you know, eat healthy and things like that. (Dennis)
Significance of high or rising PSA levels as an indicator of recurrent prostate cancer	Well, do it before treatment 'cause it [PSA level] might move and it might not. 'Cause if it moves then obviously I've got to go in for treatment. And if it doesn't move, obviously the longer it stays away then the better it is for me. And I wouldn't actually need the radiotherapy. (Sebastian)
**Theme 3: Coping with prostate cancer: Just get on with it**	
Men who wanted to return to a 'normal' life after prostate cancer were less likely to change or seek dietary/lifestyle information	Well really, to be honest, I never accept, I knew the idea of prostate leads to cancer, but I never thought that I had got it, I had it. I never think, "Oh I've got cancer." Nothing like that, no…but I was lucky enough I, I took it at the early stage, so it didn't have to develop… Just carry on with my normal life, you know. Do everything in moderation. (Fabian)
Social network and support as a facilitator to dietary and physical activity changes	…She's [sister] telling me that, you know, sugar and dairy products, that's the other things I must stop eating them things. (Laugh)...Since she told me that, I normally use skimmed milk to make my tea and I make porridge… I just try and do what she said. Less sugar, less dairy products. I just try to eat less of those things. I don't know. (Joseph)
	And it's just being in a room, with other people, that are trying to do something. There's a positive energy about being in a room… it's always better when you- well, when you walk in a pack, with people that are motivated the same way that you are. (Thomas)
**Theme 4: Ageing: Those were the days, this is me now**	
Ageing as a barrier to strenuous physical activity; adapting to changes to the body	Yeah I still do a lot of activities. Decorator, painting and decorating. I can do all that, but it's only now I, the ladder, my body is saying to me, "Don't climb the ladder much anymore."…suddenly you find the aches and pains and I can't climb the ladder anymore. (Fabian)
	Age. Cricket I am, I think last I played cricket was about maybe 15, 20 years ago and you feel as you get older you're restricted. I can't see myself running here and running there to catch a bus and that's not good for the heart. (Errol)
Ageing heightened men's awareness of their health,	… You know, it's a wise thing to change your diet, and I think I am reaching a certain age, you

For peer review only

especially in regards to their body weight	know… I think I should sacrifice and get rid of those things [starchy foods] and try and keep myself on a balanced diet where I could then chop my weight and look after myself… (Dennis)
	….I want to go to the gym soon…they reckon I'm putting on a bit too much weight so go and take off some of the weight (Laugh). (Sebastian)
**Theme 5: Autonomy: It has to be something I enjoy**	
Men who made changes out of necessity-chronic health conditions- broke their diet as they felt restricted	…but after you're diabetic you say okay, that's not good that's not good so you leave that out… Yes, so, so what I don't eating, I try to follow the rules as much as I can, but rules are meant to be broken. (Laugh). If we, if you obey all the rules all the time you miss half the fun. (Laugh) (Errol)
Preference in regards to incorporating tomatoes into diet	I eat tomatoes, but I don't like cooked ones…Fresh ones, I buy loads of them and I just squash them, cut them up and just eat them in my, eat them in bread and thing with my dinner. (Joseph)
Views on a dairy-free diet	Cut out the skimmed milk from, from my cereal, but I like my cereal, every morning. That would be difficult. I have my cereal every morning. I could cut out the cheese, but the milk. That's a difficult one for me. (Matthew)
Have to like the food to eat it, regardless of its benefits	I don't like, I can't eat the English type food most most 'cause a lot of things I don't I never eat it. They say it's good to eat, and I never eat it… (Joseph)
	…but it must be, not all fruit is palatable to me. I, I'm, I'm must like it to eat it, whether it's good for me or not I must like it. (Errol)

For peer review only

Barriers and facilitators to healthy lifestyle and acceptability of a dietary and physical activity intervention among African Caribbean prostate cancer survivors in the UK: a qualitative study

Journal:	*BMJ Open*
Manuscript ID	bmjopen-2017-017217.R1
Article Type:	Research
Date Submitted by the Author:	25-Jul-2017
Complete List of Authors:	Er, Vanessa; University of Bristol School of Social and Community Medicine,<br>Lane, Athene; University of Bristol, School of Social and Community Medicine; NIHR Biomedical Research Unit in Nutrition, Diet and Lifestyle at University Hospitals Bristol NHS Foundation Trust and the University of Bristol<br>Martin, Richard; University of Bristol, Social Medicine; NIHR Biomedical Research Unit in Nutrition, Diet and Lifestyle at University Hospitals Bristol NHS Foundation Trust and the University of Bristol<br>Persad, Raj; Bristol Urological Institute, North Bristol NHS Trust, Southmead Hospital<br>Chinegwundoh, Frank; Barts Health NHS Trust, Department of Urology; City, University of London, School of Health Sciences<br>Njoku, Victoria; Barts Health NHS Trust, Department of Urology<br>Sutton, Eileen; University of Bristol School of Social and Community Medicine
**Primary Subject Heading**:	Qualitative research
Secondary Subject Heading:	Urology, Oncology, Nutrition and metabolism, Public health
Keywords:	Prostatic neoplasms, QUALITATIVE RESEARCH, diet, physical activity, Caribbean region, survivors

1

Barriers and facilitators to healthy lifestyle and acceptability of a dietary and physical activity intervention among African Caribbean prostate cancer survivors in the UK: a qualitative study

Vanessa Er, J Athene Lane, Richard M Martin, Raj Persad, Frank Chinegwundoh, Victoria Njoku, Eileen Sutton

Corresponding author:

Dr Vanessa Er, School of Social and Community Medicine, University of Bristol, Canynge Hall, 39 Whatley Road, Clifton, Bristol, BS8 2PS, UK, E-mail: vanessa.er@bristol.ac.uk, Tel: +44 (0)117 928 7365.

Co-authors:

J Athene Lane, School of Social and Community Medicine, University of Bristol, 39 Whatley Road, Clifton, Bristol, BS8 2PS, UK. athene.lane@bristol.ac.uk

Richard M Martin, School of Social and Community Medicine, University of Bristol, 39 Whatley Road, Clifton, Bristol, BS8 2PS, UK. NIHR Bristol Nutrition Biomedical Research Unit. Level 3, University Hospitals Bristol Education & Research Centre, Upper Maudlin Street, Bristol, BS2 8AE, UK. MRC Integrative Epidemiology Unit, Oakfield House, Oakfield Grove, Bristol, BS8 2BN, UK. richard.martin@bristol.ac.uk

Raj Persad, Bristol Urological Institute, North Bristol NHS Trust, Southmead Hospital, Southmead Road, Bristol, BS10 5NB, UK. rajpersad@bristolurology.com

For peer review only

Frank Chinegwundoh, Department of Urology, Barts NHS Health Trust. The Royal London Hospital, Whitechapel Road, London E1 1BB, UK. School of Health Sciences, City, University of London. Northampton Square, London, EC1V 0HB, UK. frank.chinegwundoh@nhs.net

Victoria Njoku, Department of Urology, Barts NHS Health Trust. The Royal London Hospital, Whitechapel Road, London, E1 1BB, UK. victoria.njoku@bartshealth.nhs.uk

Eileen Sutton, School of Social and Community Medicine, University of Bristol, 39 Whatley Road, Clifton, Bristol, BS8 2PS, UK. e.sutton@bristol.ac.uk

Keywords

Qualitative research, prostatic neoplasm, diet, physical activity, Caribbean region, survivors

Word Count

5582 words

Abstract

Objectives: Diet and lifestyle may have a role in delaying prostate cancer progression, but little is known about the health behaviours of Black British prostate cancer survivors despite this group having a higher prostate cancer mortality rate than their White counterparts. We explored the barriers and facilitators to dietary and lifestyle changes and the acceptability of a diet and physical activity intervention in African Caribbean prostate cancer survivors.

Design: We conducted semi-structured in-depth interviews and used thematic analysis to code and group the data.

Participants and setting: We recruited 14 African Caribbean prostate cancer survivors via letter or at oncology follow-up appointments using purposive and convenience sampling.

Results: A prostate cancer diagnosis did not trigger dietary and lifestyle changes in most men. This lack of change was underpinned by five themes: pre-cancer diet and lifestyle, evidence, coping with prostate cancer, ageing, and autonomy. Men perceived their diet and lifestyle to be healthy and were uncertain about the therapeutic benefits of these factors on prostate cancer recurrence. They considered a lifestyle intervention as unnecessary because their prostate-specific-antigen (PSA) level was kept under control by the treatments they had received. They believed dietary and lifestyle changes should be self-initiated and motivated, but were willing to make additional changes if they were perceived to be beneficial to health. Nonetheless, some men cited advice from health professionals and social support in coping with prostate cancer as facilitators to positive dietary and lifestyle changes. A prostate cancer diagnosis and ageing also heightened men's awareness of their health, particularly in regards to their body weight.

Conclusions: A dietary and physical activity intervention framed as helping men to regain fitness and aid post-treatment recovery aimed at men with elevated PSA may be appealing and acceptable to African Caribbean prostate cancer survivors.

For peer review only

Strengths and limitations

- Our study is one of the first to explore the dietary and lifestyle practices and information seeking behaviour of African Caribbean men after a prostate cancer diagnosis in the UK.

- Views and preferences for a dietary and physical activity intervention were sought from men from diverse sociodemographic backgrounds and with different prostate cancer clinical history.

- We struggled to recruit men on active monitoring. It has been reported that Black African and Caribbean men in the UK were more likely to undergo radical treatment compared to their White counterparts,[1] perhaps due to a tendency for their prostate cancers to be managed more aggressively by clinicians.

- We did not interview men's partners but it would be of interest to explore eating habits and food choices of the family with their partners to gain a deeper understanding of the roles and responsibilities regarding food within the household.

- This study reflects only intentions in response to a dietary and physical activity intervention which may differ from that in a real intervention.

Background

Prostate cancer is the second most common cancer in men worldwide, with over 1.1 million cases diagnosed in 2012.[2] In the UK, there are over 40,000 newly diagnosed prostate cancer cases and 10,000 deaths each year.[3] It affects Black men (Black African, Black Caribbean,

For peer review only

and Other Black) disproportionately, and in England, they are at twice the risk of being diagnosed with, and dying from prostate cancer than White men of the same age.[4] The African Caribbean community in the UK is established and make up a large proportion of older adults within the Black population,[5] hence they are more likely to be affected by prostate cancer as it is strongly related to age. Awareness of prostate cancer among Black men is low, with widespread misconceptions about the methods of diagnosis and treatment.[6] Their reluctance to be tested for prostate cancer has been attributed to a lack of communication and information from health professionals, mistrust of the health-care system, perceived threats to masculinity, fear of cancer and side effects of prostate cancer treatment.[6]

Radical treatments for prostate cancer are often accompanied by adverse effects that negatively impact on men's quality of life,[7] and the prostate-specific antigen (PSA) test cannot distinguish the majority of men with indolent cancer from the minority with aggressive, fatal cancer.[8] Since diet and lifestyle are thought to play a pivotal role in prostate carcinogenesis,[9] attention has turned towards the development of dietary and lifestyle interventions to prevent the progression of prostate cancer. A dietary and lifestyle intervention may appeal to African Caribbean prostate cancer survivors if it is congruent with the health beliefs of first generation African Caribbean, as they have been found to have a preference for natural treatment and place great emphasis on food for health maintenance.[10,11] These beliefs could be linked to their memories of growing up in the Caribbean, where fresh and natural foods are in abundance and the use of herbal remedies is widespread.[10,11]

In 2007, the World Cancer Research Fund and the American Institute for Cancer Research (WCRF/AICR), which published the most comprehensive and authoritative report on environmental exposures and cancer, concluded that there is some evidence that selenium and

For peer review only

foods rich in lycopene probably decrease the risk of prostate cancer while diets high in calcium increases prostate cancer risk.[9] This was followed by the WCRF/AICR continuous update project report in 2014, which found strong evidence for a link between body fatness and increased risk of advanced prostate cancer.[12] Physical activity has been linked to a lower risk[13,14] of prostate cancer progression and mortality, and has also been shown to alleviate the adverse effects of prostate cancer treatment.[15] However, current evidence suggests that prostate cancer survivors have sub-optimal diet and lifestyle profiles[16-19] and the evidence-base to support the development of successful and conclusive interventions is lacking and of poor quality.[20] There is a high prevalence of obesity among prostate cancer survivors,[16,18,19] and the proportion of prostate cancer survivors consuming '5 a day' of fruits and vegetables in most studies was relatively low, ranging from 15.6% to 30.7%.[16,17,19] Similarly, it has been reported that over 50% of prostate cancer survivors are inactive.[16-18] Whilst there is a body of literature on the health behaviours of prostate cancer survivors, these studies were conducted in predominantly White population and in the US, with limited qualitative studies exploring the facilitators of, and barriers to dietary and lifestyle changes.[21-25]

Dietary studies of African Caribbean in the UK (mainly first-generation) found a lower energy and fat intake, and higher fruit and vegetable intake compared to the British population,[26] as they tend to adhere to a traditional Caribbean diet that is rich in complex carbohydrates; including pulses, fruits and vegetables. The impact of dietary acculturation on second generation African Caribbean in the UK was evident, with this group more likely to adopt a Westernised diet which is high in fat, sugar and low in fruits and vegetables.[27] To our knowledge, there is no qualitative study on the health behaviours of African Caribbean men after a prostate cancer diagnosis. It is important to understand the health behaviours and needs of African Caribbean prostate cancer survivors, so as to inform the development of a

For peer review only

dietary and lifestyle intervention which is both acceptable to this population and effective in improving prostate cancer outcomes. The aim of this qualitative study was to explore the facilitators and barriers to dietary and lifestyle changes, and the acceptability of a dietary and physical activity intervention among African Caribbean prostate cancer survivors to inform the development of a high-quality diet and physical activity randomised controlled trial.

Methods

Study population and ethical approval

We recruited men aged 18 and above who self-identified as African Caribbean, and had a clinically–confirmed prostate cancer diagnosis from two large cities in the UK by invitation letter through their consultants and in-person at oncology follow-up clinics. We used purposive sampling to select men across a range of ages, socioeconomic positions, marital status and treatment types to obtain a representative sample.[28] These factors have been identified in the literature to influence dietary and lifestyle changes after a prostate cancer diagnosis.[29] As a result of slow recruitment at the first site and time constraints – this study was conducted as part of a doctoral degree - we used convenience sampling[28] and focused our recruitment on another site with larger African Caribbean community. We aimed to recruit between 15 and 20 participants. The sample size was expected to obtain sufficient data to detect possible themes based on previous research[29,30] and advice from experienced qualitative researchers.[31-33] We were unable to recruit men on active monitoring. Nonetheless, no new themes were emerging from the data after 14 participants had been interviewed. Ethical approval was provided by the Greater Manchester West Research Ethics Committee (13/NW/0878).

In-depth interviews

For peer review only

All participants provided their written informed consent to take part in the study, for their interviews to be audio-recorded and quotes to be published. A researcher (VE) carried out in-depth, semi-structured interviews with participants at their homes in between 2014 and 2015. Interviews lasted between 45 minutes and 1 hour 40 minutes and participants received a £15 supermarket voucher at the end of the interview as a thank you for taking part. VE presented herself as a student who would like to know the participants' view on diet and prostate cancer as part of her studies. Although she has a Masters degree in nutrition, she did not reveal this to the participants. Her interest in researching diet and lifestyle of people from minority ethnic groups is due to her experience of growing up in Malaysia, a multiracial and multicultural country, and was surprised to discover that despite being disproportionately affected, there was a lack of research on the health behaviours of Black men in the UK diagnosed with prostate cancer.

VE used an interview topic guide (**Supplementary Material 1**) to direct discussions on men's dietary and lifestyle practices before and after a prostate cancer diagnosis, the reasons for dietary and/or lifestyle changes (or lack of), sources and views on dietary and lifestyle information, and views on a proposed dietary and physical activity intervention for prostate cancer survivors, which consists of increasing tomato intake, a dairy-free diet and 30 minute of brisk walking a day. The topic guide was informed by the literature, reviewed by a public and patient involvement group, and was refined twice based on preliminary findings from early interviews to include topics on body weight, familiarity with soya products, and tomatoes being a feature in the Caribbean diet.

Data analysis

For peer review only

The interviews and data analysis were carried out concurrently.[34] This was an iterative process such that salient issues or topics raised in early interviews could be explored or follow-up in subsequent interviews. All interviews were anonymised, transcribed verbatim and analysed using thematic analysis[35] with the aid of a qualitative data analysis computer software package (NVivo 10). The researcher (VE) used an inductive approach to code the data in 'chunks' to keep the context where the data originated, and developed an initial coding frame.[35] A portion of transcripts was coded independently by another researcher (ES) to ensure coding was consistent and was a balanced representation of participants' views. Throughout the coding process, extracts of data across the dataset were compared to check for similarities and differences.[35] The coding framework was refined and revised by VE and ES as the analysis progressed. We identified, grouped and conceptualised patterns within and across the dataset which are salient to the participants into themes.[35] Field notes and analytic memos written by VE were used to explore how codes and themes related to each other and aided the conceptualisation of key themes. VE and ES discussed any discrepancies and finalised the themes. All names presented here refer to pseudonyms.

Results

The characteristics of the participants are presented in **Table 1**. At the time of interview, the average age of participants was 69.6 years. Most of the men were married or living with a partner; diagnosed with prostate cancer less than five years at the time of the interview; and had radiotherapy and adjuvant hormone therapy as treatment. With the exception of five men, the rest of the participants were retired. Most men have or previously had semi-routine or routine occupation.

For peer review only

Table 1 Characteristics of participants

Pseudonym	Age	Marital status	Years since diagnosis	Cancer stage	Treatment	Co-morbidities
Aaron	79	M/P	≤1	Localised	Not started[a]	Hypertension, Gastro-oesophageal reflux disease
Dennis	56	M/P	≤1	Localised	Not started[a]	Hypertension
Jonah	75	M/P	>10	Locally advanced	RT/HT	-
Fabian	73	M/P	>1 and <5	Locally advanced	RT/HT	Type 2 Diabetes
Colton	77	M/P	>10	Locally advanced	RT/HT	Hyperthyroidism
Albert	65	M/P	≥5 & <10	Localised	RT/HT	Hay fever
Jamal	70	S/W	>1 and <5	Locally advanced	RT/HT	-
Matthew	80	S/W	>10	Locally advanced	RT/HT	Type 2 Diabetes
Irvin	79	S/W	>1 and <5	Localised	RT/HT	-
Errol	76	S/W	≤1	Locally advanced	RT/HT	Type 2 Diabetes
Shaun	68	M/P	≤1	Localised	RT/HT	Type 2 Diabetes, Asthma
Joseph	63	M/P	≤1	Localised	Prostatectomy	-
Sebastian	61	M/P	≤1	Localised	Prostatectomy	-
Thomas	52	M/P	≤1	Localised	Prostatectomy	-

RT/HT: radiotherapy and adjuvant hormone therapy. M/P: Married or living with a partner. S/W: Single or widowed.
[a] Had not started treatment at the time of interview.

Five themes were identified from the analysis which captured the barriers and facilitators to dietary and lifestyle changes, the rationale for seeking (or not) dietary and lifestyle information, and men's views on the target population, timing, and acceptability of a proposed dietary and physical activity intervention. Additional quotes relating to each theme are provided in **Supplementary Material 2.**

Theme 1: Pre-cancer diet and lifestyle

w only

Most men who perceived their diet to be healthy before a prostate cancer diagnosis ate as usual. Some men had already made positive dietary changes prompted by other health problems such as diabetes, while others questioned what changes could be made if they were already eating healthily.

> *Well, but erm - change to what? (Laugh) What do you change to, in those circumstances? If you eat, you know, if you eat healthy food, meat, vegetables, salads, fruits, what do you change to? (Jonah)*

Thus, most of the men did not seek further dietary information after a prostate cancer diagnosis as they perceived themselves as having a healthy lifestyle and knowing what constitutes a healthy diet. When men were asked for their views on a dietary intervention which would require them to eat more tomatoes and cut out dairy products, they were amenable to it because it complemented their existing diet. They explained that tomatoes are commonly consumed as part of a traditional Caribbean diet, while dairy products only account for a small fraction of it. Similarly, a proposed physical activity intervention which involves brisk walking for 30 minutes a day was considered achievable, with several men already doing it in their daily lives.

Men who perceived themselves to be active before prostate cancer also did not think they needed to increase their physical activity. For men, being active meant being up on their feet and 'doing things', but they advocated moderation. As one participant (Errol) noted: *"I do the exercise in my work. 'Cause when you're a builder, you don't sit, you don't do nothing, you have to move around all the time, you know. So that to me enough exercise."* A few men wanted to be more active but they were impeded by health conditions or sports injuries. Conversely, men who made changes acknowledged that their diet and lifestyle before

For peer review only

prostate cancer could have been better and a prostate cancer diagnosis acted as a catalyst for the change.

> *…But since I was diagnosed, er, we just, I would say about, the beef probably come out, we cut down beef a bit, you know? … they always say too much red beef is not good for you and that. So we just cut it down, you know? (Albert)*

Theme 2: Evidence: link between diet, lifestyle, and prostate cancer

Men's views on the causes of cancer varied, ranging from genetic factors, fate or chance, stress (not enough rest), diet and lifestyle to food production (chemicals). However, the majority of men were aware that Black men have higher risk of prostate cancer, and some described it as an 'old man's complaint'. A few men cited a lack of evidence that prostate cancer is linked to diet and lifestyle to support their view. This was contrasted with men's belief that smoking increases the risk of cancer due to widely established evidence that it causes lung cancer.

> *…up until now they haven't come out with err, err, anything positive to say, "This is the result, this is the main reason why black men from the Caribbean or West Africa have a, how I say, the most higher rate."…So I don't think that I am in any position to think of, because of our diet or because of what we eat, do you understand? (Dennis)*

Men who believed that prostate cancer is not connected to diet were less likely to seek dietary information. This was partly due to their mistrust of the dietary messages from media which they regarded as conflicting. They preferred receiving information from health professionals who they regarded as experts and a trusted source of health information, and that had a positive influence on their health behaviour. For example, one man (Colton) started eating tomatoes and drinking pomegranate juice because his clinicians informed him that it could be

For peer review only

beneficial for prostate cancer: *"she [dietician] said with, with the prostate cancer that I've got I should eat a lot of tomato. Tomato is good for the prostate cancer. My doctor before, Doctor M, told me that if I drink pomegranate juice it's a little bit helpful as well…That's what I buy, we buy, things, we buy pomegranate juice."* However, only a few men reported that they received dietary or lifestyle advice from health professionals, with some explaining that their doctor was uncertain about the effectiveness of diet and lifestyle on prostate cancer.

There was however a small number of men who believed that diet and lifestyle are important for cancer prevention, but they did not think diet and lifestyle have any impact on cancer progression, particularly if their treatment was effective: *"So, so what the difference that it make to, it's not going to affect me now because I'm, I've [passed that stage] with the prostate cancer" (Albert).* Consequently, men suggested that a dietary and physical activity intervention should be offered before treatment, as the effect of the intervention would not be confounded by the treatment, and if the intervention works, treatment would not be necessary. The significance of PSA became apparent when men explained their views, as a high or rising PSA level after treatment was perceived as indicative of recurrent prostate cancer.

> *Well, do it before treatment 'cause it [PSA level] might move and it might not. 'Cause if it moves then obviously I've got to go in for treatment. And if it doesn't move, obviously the longer it stays away then the better it is for me. And I wouldn't actually need the radiotherapy. (Sebastian)*

Accordingly, they believed that interventions should be targeted at men who have a high PSA level and were more likely to adopt dietary or lifestyle advice if it was shown to reduce or keep PSA level low.

For peer review only

Theme 3: Coping with prostate cancer: just get on with it

Men had differing approaches to coping with prostate cancer which influenced whether they made positive changes to their diet and lifestyle. Those who were sanguine about prostate cancer tended to 'take it as it comes' and thus less likely to change their diet and lifestyle and were not interested in gaining further dietary and lifestyle information. Similarly, men who considered their cancer as treated just wanted to move on and not dwell upon it further.

> *Well me don't really want to get no more information, because so far I'm not living with the thing again. So I don't really want to get no more information, I just try to do, the, my best, try to help myself in many ways. (Jamal)*

These approaches were directly contrasted with those of men who were actively seeking ways to alleviate the side effects they experienced and wanting to be fit again. Thomas spoke about exercise as a way of making himself feel better as it felt like he was doing something for his body, rather than relying on medication for depression or pain: "*If there's something that you need, to motivate yourself and exercise, might trigger it off. I'm not sure, but I know that sitting down, taking tablets, isn't going to do it, or not even diet, to that point.*"

It became evident that social networks and support were important for men in coping with prostate cancer, and facilitated positive dietary and lifestyle changes. Partners, family and friends acted as a 'change agent' to enable these changes. The role of partner or wife in initiating dietary changes and encouraging men to live healthier is highlighted in this comment.

> *… more of less it's the wife that is leading the process [dietary change] really. (Laugh) Yeah, she's the one doing the shopping, she's the one providing the food. And now*

For peer review only

she's, she's going and, and do all the shopping and buy whatever she thinks that's good for our health. (Dennis)

Several men commented on the importance of social support to stay physically active. They were very positive about group exercise citing shared purpose and motivation as an attraction to them, as one participant (Matthew) commented: *"what group exercises do is to encourage you and encourage others to follow. That is why I used to like going to the gym, 'cause you had people there who were there for the same purpose."*

Whether men made dietary and lifestyle changes was also dependent on their priorities and concerns after cancer treatment. Men typically experienced side effects from treatments including incontinence and erectile dysfunction. Thus, coping and adapting to these side effects were their main priorities. Primarily, incontinence was reported as a barrier to physical activity.

I want to go to the gym soon, but like I said, because I'm so wet I won't have – I'm hoping that it will ease up a little bit so I can get to do something else….(Sebastian)

Loss of income because of a prostate cancer diagnosis could also have a knock-on effect on opportunities for physical activity. Sebastian who was self-employed had financial worries and his main priority was to return to work, for without an income, he could not afford to pay for gym membership.

Theme 4: Ageing: those were the days, this is me now

Most men used to play sports and acknowledged the health benefits of physical activity, but old age was the most commonly cited reason for not doing strenuous physical activity. For

For peer review only

men who had retired, life after work was portrayed as a time to rest (slow down) and for leisure. Most men (aged 70 and above) viewed themselves as too old to be playing sports and gentle exercise as safer and more appropriate for their age. Therefore, a brisk walking intervention was perceived as safe and acceptable by men in the study.

> *I'm too old to go to the gym man. I don't want to go to the gym to get a heart attack. (Laughter) Because I don't know, I don't go there [to lift up] these big great things, I go…on the treadmill. (Laugh) I used to go on it but I don't want to do all these type of things now. (Jamal)*

Men made frequent references to their body when describing their physical limitations. They also talked about having to accept and adapt to the changes in their body as they aged. As one participant (Joseph) noted: *"Age is against me now (laugh). I don't feel much pain, but you can tell on the body. Your body can't cope with that. I know it can't cope with it anymore."*

Younger men (under 65 years old) however had no qualms about performing higher intensity physical activity. For Thomas, being back in the gym and having the ability to do the exercises just as before, was a sign of recovery - "*erm- because I've been in a gym before, it's going back to an old friend, and actually familiarising yourself with it, and being told- and telling yourself that you- you're back on the road to recovery…To be back in that gym environment again, it- it was lovely…*" Similarly, Dennis who had a prostate biopsy before the interview hoped to continue playing cricket for his club - *"Well, I used to play every Sunday…but since I had the operation I haven't played…when I am feeling healthy and okay I will"*.

For peer review only

On the other hand, men had more awareness of their health as they aged because they perceived that they were more susceptible to illness, so having a healthy diet and lifestyle became more relevant to them. For the men in this study, ageing also shifted the focus from providing for the family to taking care of themselves. There was also a perception espoused by men that getting older made one wiser and thus more sensible. Some men noticed that they put on weight more easily as they aged, mainly because they were less active than before. That prompted men to eat less, cut down food portion sizes, alcohol intake or exercise more.

> *Yeah, that's why I said don't want to eat fatty things. I don't want to be bloated out, put it that way…Yeah, yeah, I don't want to get big, fat and clumsy man, where I can't move about and things like that. So I, I watch, I know what I'm eating. (Jamal)*

The younger men who experienced side effects from prostate cancer treatments, especially incontinence and fatigue, and thus unable to perform strenuous physical activity also expressed concerns about their weight and wanted to regain fitness to cope with these side effects.

> *… she [trainer] said, "You've got a bigger belly than you had before, and if you want- what do you want to get out of this?" And I said, "I'd like to probably get my shape back, and just lose a little bit of weight…" (Thomas)*

Theme 5: Autonomy: it has to be something I enjoy

Overall, men believed that an individual has to be personally motivated to make dietary and lifestyle changes so that they would be maintained long-term. Men who made changes to their diet and lifestyle out of necessity due to co-morbidities such as diabetes, felt restricted and thus sometimes broke the rules. Although the proposed dietary intervention which

For peer review only

required men to increase intake of tomatoes was acceptable for most men, they expressed different preferences in regards to taste and ways of incorporating tomatoes in their diet. A few men favoured whole tomatoes over lycopene supplement (tablets) because they were suspicious of its content and production process.

> *It would be easier, but- but, I mean, it's- yeah, it would be easier, without a doubt. If you could take a tomato tablet that does it, but then it's what goes into making the tablet. With a tomato, a fresh tomato, you know what you're getting, don't you? (Thomas)*

Similarly, men in general were amenable to a dairy-free diet but they had reservations about substituting dairy products with soya, because they were unfamiliar with soya products and perceived dairy substitutes as unnecessary due to a low dairy intake. Nevertheless, there were a few men who were hesitant to give up milk which they usually consumed with breakfast cereals or in porridge. Some men also commented that they have to enjoy or like the food to eat it, regardless of its health benefits. One man rejected the idea of eating tomatoes every day despite being told of the potential protective effect of tomatoes from prostate cancer – *"No I won't eat all that…normally I use one for the salad - if I make salad – [I wouldn't want to do]… You know? I don't eat every day…I don't feel like doing it, and I don't want to do it. (Laugh)"* (Irvin).

Finally, the majority of the men interviewed preferred to walk on their own instead of taking part in a group exercise, even though they recognised the support and motivation that it could provide. Men explained that each person has his own schedule and routine, thus it is easier to walk on their own and at a pace that suited them.

For peer review only

Na, because again I have to, they want, when I'm ready to do it they won't be ready, that's the problem. That's why I have to do my own. (Albert)

Discussion

Most men in our study did not change their diet and lifestyle after a prostate cancer diagnosis. The lack of change was underpinned by five key themes: pre-cancer diet and lifestyle, evidence, coping with prostate cancer, ageing, and autonomy. The implications for research and practice are described below.

Design of dietary and lifestyle intervention for prostate cancer survivors

The proposed dietary and physical activity intervention was acceptable to men in this study, but they suggested it should be aimed at men with an elevated or rising PSA level, including those who are on active monitoring. The majority of dietary and lifestyle interventions for cancer survivors have been conducted after treatment with the aim of improving general health, and quality of life, but this finding suggests that men may be more interested in the clinical effect of interventions. Consequently, men perceived the proposed intervention as unnecessary because their PSA level was kept under control by the treatments they had received, which supports the findings of a previous study[29] and highlights the significance of using PSA as an outcome measure, despite criticism of its usefulness as a surrogate end-point in prostate cancer trials.[36,37] It also illustrates the challenges of informing men that dietary or lifestyle factors may act on prostate cancer independently of PSA.

This study also showed that men valued autonomy in their food and lifestyle choices and they might change their diet and lifestyle if it was deemed necessary and beneficial for health. Similar studies examining healthy eating using gender theory found that resistance among

For peer review only - http://bmjopen.bmj.com/site/about/guidelines.xhtml

men was linked to masculine ideals of autonomy and rationality.[38] The authors suggest that healthy eating initiatives and materials could be tailored to men by emphasising personal choices and responsibility.[38] Most men in our study preferred individual over group exercise, which differed from previous studies reporting that men enjoyed the opportunity to socialise when exercising.[25,39] Nonetheless, men were willing to participate in weekly group exercise provided that a time and place that is convenient for all could be found. Therefore, either a choice of home-based intervention only or in combination with weekly supervised group exercise may appeal to African Caribbean prostate cancer survivors.

Provision of dietary and lifestyle advice and support

Evidence from existing literature suggests that most men were uncertain about the role of diet and lifestyle in preventing prostate cancer progression or recurrence, but a strong belief in cancer-diet relationship was a predictor of dietary changes.[21-23,29,30] As with previous studies,[21,24,25,29,30] our findings suggest that advice from health professionals can strengthen men's beliefs on the relationship of prostate cancer with diet and lifestyle, influencing men to make positive lifestyle changes. Most men in our study did not recall receiving dietary or lifestyle information from health professionals, perhaps due to their hesitance on providing dietary and lifestyle advice because of a lack of evidence on diet and prostate cancer and awareness of relevant guidelines for cancer survivors.[29,40] Nonetheless, it is becoming clear that obesity is associated with higher risk of advanced and fatal prostate cancer,[12] and health professionals could potentially play a role in influencing men's health behaviour.

Awareness of supporting services was low among our participants. The UK National Cancer Survivorship Initiative (NCSI) recommend a 'recovery package' which consists of a combination of interventions, including a patient education and support event (health and

For peer review only

wellbeing clinic) to be offered to all cancer survivors.[41] A report by the London Cancer Alliance found that there was no standardised definition or structure for health and wellbeing clinics in their partner NHS trust providers.[42] Therefore, more research is needed in designing and evaluating the referral pathways for healthy lifestyle services, and developing a coherent model for health and wellbeing clinics.

Active ageing and body weight

In line with previous research,[43,44] misconceptions about the harms or risks of strenuous physical activity in old age such as heart attack and feeling breathless were shared by men in this study. Older men also felt that going to the gym was incompatible with their age as for them it was typically associated with young people ‘pumping iron’. Although there has been much effort in the UK and developed countries to promote active ageing, this shows that further work is needed to challenge prevailing social norms on ageing and physical activity to ensure men stay active as they age. Older African Caribbean prostate cancer survivors should be informed that resistance exercise is beneficial to their health especially for preventing loss of muscle mass in men on androgen deprivation therapy.

Ageing or being diagnosed with prostate cancer also heightened men’s awareness of their health and prompted several men to make positive changes to their diet and lifestyle for maintenance of general health. Concerns about body weight among older men and prostate cancer survivors have been reported in several studies.[22,29,38] In this study, men wanted to maintain a healthy weight to avoid having a ‘big belly’. This differs from previous studies which found that men lose weight for health purposes rather than for appearance (body image).[45,46] Men in this study watched their weight by eating smaller portion sizes, having light meals (less starchy food) and increasing their physical activity, contradicting previous

For peer review only

studies which found that men preferred to lose weight through exercising because it is regarded as a masculine pursuit,[45] while 'dieting' practices such as increasing fruits and vegetable intake and reducing food portion sizes have been perceived to be feminine.[46,47]

Younger men also expressed interest in losing weight and regaining fitness after a period of inactivity due to side-effects of treatment. Similar to previous studies, men who experienced urinary incontinence were unsure about the type and intensity of physical activity that they could perform for fear of exacerbating their condition.[24,25,39] Thus, a supervised physical activity intervention would appeal to men to help them to lose weight and alleviate the side effects of treatments. A physical activity intervention could also be framed as a way for men to take charge of their recovery for better health.

Strengths and limitations

Our study is one of the first to explore the dietary and lifestyle practices and information seeking behaviour of African Caribbean men after a prostate cancer diagnosis in the UK. Additionally, views and preferences for a dietary and physical activity intervention were sought from men to inform the design of a tailored intervention. It included men from diverse sociodemographic backgrounds and with different prostate cancer clinical history.

We struggled to recruit men on active monitoring. It has been reported that Black African and Caribbean men in the UK were more likely to undergo radical treatment compared to their White counterparts,[1] perhaps due to a tendency for their prostate cancers to be managed more aggressively by clinicians.[48] Additionally, the proportion of men on active monitoring is relatively low – 12% in UK in 2009[48] - compared to other prostate cancer treatments. We did not interview men's partners. It would be of interest to explore eating habits and food

For peer review only

choices of the family with their partners to gain a deeper understanding of the roles and responsibilities regarding food within the household, and contrast with the diet of single/widowed men who live alone.

There was an indication that men preferred to consume tomatoes over a lycopene supplement, which requires further investigation. Furthermore, questions on format of delivery (telephone-based, print-based, supervised physical activity) and ways of monitoring adherence (food diary, pedometers) were not explored in all interviews, precluding comparisons between men. In future research, clear and simple visual aids could be used to present and facilitate discussions on the proposed intervention. Finally, this study reflects only intentions in response to a dietary and physical activity intervention; this may differ from that in a real intervention.

Conclusions

A prostate cancer diagnosis did not trigger dietary and lifestyle changes in this group of men who mostly perceived their diet and lifestyle to be healthy and were uncertain about the therapeutic benefits of diet and lifestyle on prostate cancer recurrence. They considered an intervention as unnecessary because their PSA level was kept under control by the treatments they had received. Men believed dietary and lifestyle changes should be self-initiated and motivated but were willing to make additional changes if they were perceived to be beneficial to health. Nonetheless, some men cited advice from health professionals and social support in coping with prostate cancer as facilitators to positive dietary and lifestyle changes. Furthermore, a prostate cancer diagnosis and ageing heightened men’s awareness of their health, particularly in regards to their body weight. Therefore, a dietary and physical activity intervention which enhances men’s autonomy, framed as helping men to regain fitness and

For peer review only

aid post-treatment recovery, and is aimed at men with elevated PSA may be appealing and acceptable to African Caribbean prostate cancer survivors.

Declarations

Contributing statement

VE, ES, JAL, and RMM conceived the study; VE, ES, FC, RJ and VN contributed to the design and conduct of the research, and acquisition of data; VE and ES analysed the data; VE, ES, RMM and JAL wrote the first draft of the paper; ES, JAL, and RMM provided supervision; VE, ES, JAL, and RMM have primarily responsibility for final content. All authors were involved in revising the paper, and read and approved the final version of the paper.

Acknowledgement

The authors would like to thank all participants for sharing their views and experience; Dr. Mona Jeffreys and the Bristol Nutrition BRU prostate patient and public involvement group for their input into the study design and documents; and all staff who had helped with the recruitment of participants, especially Dr. Karen Tipples.

Disclaimers

The views expressed herein are those of the authors and do not necessarily reflect those of Cancer Research UK, the NHS, the NIHR, or the Department of Health.

Competing interest

All authors have completed the ICMJE uniform disclosure form at www.icmje.org/coi_disclosure.pdf and declare: no support from any organisation for the

For peer review only

submitted work; no financial relationships with any organisations that might have an interest in the submitted work in the previous three years; no other relationships or activities that could appear to have influenced the submitted work.

Funding

This work was supported by the National Institute for Health Research (NIHR) Bristol Nutrition Biomedical Research Unit. The unit is a partnership between University Hospitals Bristol NHS Foundation Trust and the University of Bristol. VE was a recipient of a 3-year PhD studentship at the Bristol Nutrition Biomedical Research Unit from 2012-2015. RMM and JAL are recipients of CRUK programme funding (C18281/A19169).

Data sharing statement

The datasets generated and/or analysed during the current study are not publicly available as participants may be identifiable, but are available from the corresponding author on reasonable request.

Ethics approval and consent to participate

Ethical approval was provided by the Greater Manchester West Research Ethics Committee (13/NW/0878). All participants provided their written informed consent to take part in the study.

For peer review only

References

1. Evans S, Metcalfe C, Patel B, et al. Clinical presentation and initial management of Black men and White men with prostate cancer in the United Kingdom: the PROCESS cohort study. *Br J Cancer* 2010;102(2):249-54.
2. Ferlay J, Soerjomataram I, Dikshit R, et al. Cancer incidence and mortality worldwide: sources, methods and major patterns in GLOBOCAN 2012. *Int J Cancer* 2015;136(5):E359-86.
3. Office for National Statistics. Cancer incidence and mortality in the United Kingdom 2008-2010 http://webarchive.nationalarchives.gov.uk/20151013220531/http://www.ons.gov.uk/ons/rel/cancer-unit/cancer-incidence-and-mortality/2008-2010/index.html2012 [accessed July 2017].
4. Lloyd T, Hounsome L, Mehay A, et al. Lifetime risk of being diagnosed with, or dying from, prostate cancer by major ethnic group in England 2008-2010. *BMC Med* 2015;13:171.
5. Centre for Policy on Ageing (UK). The ageing of the ethnic minority populations of England and Wales: findings from the 2011 census. UK, 2013.
6. Pedersen VH, Armes J, Ream E. Perceptions of prostate cancer in Black African and Black Caribbean men: a systematic review of the literature. *Psychooncology* 2012;21(5):457-68.
7. Bangma CH, Roemeling S, Schroder FH. Overdiagnosis and overtreatment of early detected prostate cancer. *World J Urol* 2007;25(1):3-9.
8. Lilja H, Ulmert D, Vickers AJ. Prostate-specific antigen and prostate cancer: prediction, detection and monitoring. *Nat Rev Cancer* 2008;8(4):268-78.
9. World Cancer Research Fund, American Institute for Cancer Research. Food, nutrition, physical activity, and the prevention of cancer: a global perspective. Washington DC: AICR 2007.
10. Brown K, Avis M, Hubbard M. Health beliefs of African-Caribbean people with type 2 diabetes: a qualitative study. *Br J Gen Pract* 2007;57(539):461-69.
11. Nanton V, Dale J. 'It don't make sense to worry too much': the experience of prostate cancer in African-Caribbean men in the UK. *Eur J Cancer Care* 2011;20(1):62-71.
12. World Cancer Research Fund International, American Institute for Cancer Research. Diet, nutrition, physical activity and prostate cancer. Continuous update project: World Cancer Research Fund, 2014.
13. Kenfield SA, Stampfer MJ, Giovannucci E, et al. Physical activity and survival after prostate cancer diagnosis in the health professionals follow-up study. *J Clin Oncol* 2011;29(6):726-32.
14. Bonn SE, Sjolander A, Lagerros YT, et al. Physical activity and survival among men diagnosed with prostate cancer. *Cancer Epidemiol Biomarkers Prev* 2015;24(1):57-64.
15. Gardner JR, Livingston PM, Fraser SF. Effects of exercise on treatment-related adverse effects for patients with prostate cancer receiving androgen-deprivation therapy: a systematic review. *J Clin Oncol* 2014;32(4):335-46.
16. LeMasters TJ, Madhavan SS, Sambamoorthi U, et al. Health behaviors among breast, prostate, and colorectal cancer survivors: a US population-based case-control study, with comparisons by cancer type and gender. *J Cancer Surviv* 2014;8(3):336-48.
17. Blanchard CM, Courneya KS, Stein K. Cancer survivors' adherence to lifestyle behavior recommendations and associations with health-related quality of life: Results from the American Cancer Society's SCS-II. *J Clin Oncol* 2008;26(13):2198-204.

For peer review only

18. Coups EJ, Ostroff JS. A population-based estimate of the prevalence of behavioral risk factors among adult cancer survivors and noncancer controls. *Prev Med* 2005;40(6):702-11.
19. Rogers LQ, Courneya KS, Paragi-Gururaja R, et al. Lifestyle behaviors, obesity, and perceived health among men with and without a diagnosis of prostate cancer: A population-based, cross-sectional study. *BMC Public Health* 2008;8:43-58.
20. Hackshaw-McGeagh LE, Perry RE, Leach VA, et al. A systematic review of dietary, nutritional, and physical activity interventions for the prevention of prostate cancer progression and mortality. *Cancer Causes Control* 2015;26(11):1521-50.
21. Satia JA, Walsh JF, Pruthi RS. Health behavior changes in White and African American prostate cancer survivors. *Cancer Nurs* 2009;32(2):107-17.
22. Coa KI, Smith KC, Klassen AC, et al. Exploring important influences on the healthfulness of prostate cancer survivors' diets. *Qual Health Res* 2015;25(6):857-70.
23. Mroz LW, Chapman GE, Oliffe JL, et al. Prostate cancer, masculinity and food. Rationales for perceived diet change. *Appetite* 2010;55(3):398-406.
24. Craike MJ, Livingston PM, Botti M. An exploratory study of the factors that influence physical activity for prostate cancer survivors. *Support Care Cancer* 2011;19(7):1019-28.
25. Keogh JW, Patel A, MacLeod RD, et al. Perceived barriers and facilitators to physical activity in men with prostate cancer: possible influence of androgen deprivation therapy. *Eur J Cancer Care (Engl)* 2014;23(2):263-73.
26. Vyas A, Greenhalgh A, Cade J, et al. Nutrient intakes of an adult Pakistani, European and African-Caribbean community in inner city Britain. *J Hum Nutr Diet* 2003;16(5):327-37.
27. Sharma S, Cade J, Riste L, et al. Nutrient intake trends among African-Caribbeans in Britain: a migrant population and its second generation. *Public Health Nutr* 1999;2(4):469-76.
28. Ritchie J, Lewis J, Nicholls CM, et al. Qualitative research practice: A guide for social science students and researchers. London: Sage 2013.
29. Avery KN, Donovan JL, Horwood J, et al. The importance of dietary change for men diagnosed with and at risk of prostate cancer: a multi-centre interview study with men, their partners and health professionals. *BMC Fam Pract* 2014;15:81.
30. Horwood JP, Avery KN, Metcalfe C, et al. Men's knowledge and attitudes towards dietary prevention of a prostate cancer diagnosis: a qualitative study. *BMC Cancer* 2014;14:812.
31. Baker SE, Edwards R, Doidge M. How many qualitative interviews is enough?: Expert voices and early career reflections on sampling and cases in qualitative research. 2012
32. Guest G, Bunce A, Johnson L. How many interviews are enough? An experiment with data saturation and variability. *Field methods* 2006;18(1):59-82.
33. Hennink MM, Kaiser BN, Marconi VC. Code saturation versus meaning saturation: How many interviews are enough? *Qual Health Res* 2017;27(4):591-608.
34. Glaser B, Strauss, A. The discovery of grounded theory: strategies for qualitative research. New York: Aldine 1967.
35. Braun V, Clarke V. Using thematic analysis in psychology. *Qualitative Research in Psychology* 2006;3(2):77-101.
36. Thompson IM. PSA: a biomarker for disease. A biomarker for clinical trials. How useful is it? *J Nutr* 2006;136(10):2704S.
37. Collette L. Prostate-specific antigen (PSA) as a surrogate end point for survival in prostate cancer clinical trials. *Eur Urol* 2008;53(1):6-9.

For peer review only

38. Gough B, Conner MT. Barriers to healthy eating amongst men: a qualitative analysis. *Soc Sci Med* 2006;62(2):387-95.
39. Bourke L, Doll H, Crank H, et al. Lifestyle Intervention in Men with Advanced Prostate Cancer Receiving Androgen Suppression Therapy: A Feasibility Study. *Cancer Epidemiol Biomarkers Prev* 2011;20(4):647-57.
40. Sutton E, Hackshaw-McGeagh LE, Aning J, et al. The provision of dietary and physical activity advice for men diagnosed with prostate cancer: a qualitative study of the experiences and views of health care professionals, patients and partners. *Cancer Causes Control* 2017;28(4):319-329.
41. Department of Health (England), Macmillan Cancer Support, NHS Improvement (England). Living with & beyond cancer: taking action to improve outcomes. London, UK, 2013.
42. London Cancer Alliance. Health and wellbeing events mapping. UK, 2015.
43. Grant BC. 'You're never too old': beliefs about physical activity and playing sport in later life. *Ageing and Society* 2001;21:777-98.
44. Cousins SOB. Exercise, aging, and health: Overcoming barriers to an active old age. Philadelphia, PA: Taylor & Francis 1998.
45. De Souza P, Ciclitira KE. Men and dieting: A qualitative analysis. *J Health Psychol* 2005;10(6):793-804.
46. Gough B. 'Real men don't diet': an analysis of contemporary newspaper representations of men, food and health. *Soc Sci Med* 2007;64(2):326-37.
47. Jensen KO, Holm L. Preferences, quantities and concerns: socio-cultural perspectives on the gendered consumption of foods. *Eur J Clin Nutr* 1999;53(5):351-59.
48. Hounsome L, Iles M, Verne J. Treatment routes in prostate cancer. UK: The National Cancer Intelligence Network, 2012.

For peer review only

Supplementary Material 1

Diet and Lifestyle after Prostate Cancer Diagnosis

Topic Guide

Aims and Objectives

The aim of this study is to gather the evidence for dietary and lifestyle changes in prostate cancer survivors to inform a dietary and lifestyle intervention for randomised trials. This will involve exploring:

- Motivators and barriers to changes in dietary and lifestyle behaviours
- Sources of information on diet and lifestyle
- Experience and views of dietary and lifestyle interventions
- Preferences for intervention (delivery format)

For peer review only

Introduction

- Thank you for participating
- Introduce self and Uni Bristol
- Introduce the study: what is it about and why (limited information)
- Talk through key points:
  - length of interview
  - interview like a discussion, but will cover key topics
  - no right or wrong answers
  - participation is voluntary, rights to withdraw
  - recording interview (concentrate on what you are saying, accuracy)
- Confidentiality and anonymity, how findings will be reported
- Thank you payment
- Questions?
- Happy to proceed? Sign consent form

START RECORDING

For peer review only - http://bmjopen.bmj.com/site/about/guidelines.xhtml

University of BRISTOL

University Hospitals Bristol NHS
NHS Foundation Trust

1. Background and medical history

- **Age; household** (live alone or with others)
  - relationship with
  - married/widowed/single/partner

- **Place of birth; religion**
  - if born outside of UK, where? how long living in the UK
  - follow any religion

- Main **daytime activity**
  - working or not: details

- **Other interests/activities**

- **Prostate cancer**
  - when diagnosed
  - treatment(s)

- **Overall health**
  - health conditions that **may affect diet/lifestyle**
    e.g. diabetes, heart problems, high blood pressure

- Importance and practicalities attached to **food and physical activity**
  - family/culture (who does the cooking/food purchase)
  - involvement in sports/food-related organisation (e.g. gym, community allotment)

2. Diet and lifestyle behaviours

- **Eating habits before diagnosis**
  - fruit and veg, dairy, meat, high fat/sugar
  - alcoholic drinks, juice
  - supplements, herbal products, others
- **Remained the same**?

IF NO

  - **Describe changes**
    - adding/removing/change dietary pattern (overhaul)
  - **When** change
  - **Reasons** for change
    - perception of diet before diagnosis
    - beliefs (role of diet in prostate cancer)
    - social and/or family pressure

For peer review only - http://bmjopen.bmj.com/site/about/guidelines.xhtml

University of BRISTOL

University Hospitals Bristol NHS
NHS Foundation Trust

- **Reasons** for change *(continued)*
  - control
  - fear of recurrence/improve overall wellbeing
- **Maintenance**

- **Motivators and facilitators**
  - wife/partner/family (food purchase and cooking)
  - advice, information (advice from Health Professionals)
  - benefits to health/prostate cancer

- **Barriers (especially for maintaining changes)**
  - cost, time
  - risk to health/prostate cancer
  - treatment side-effects/fatigue/general health

- **Physical activity/exercise before diagnosis**
  - Types
  - how often, duration
  - where (gym, local leisure centre, parks)
- **Remained the same**?

IF NO
  - Describe changes
  - When change
  - Reasons for change

NB: For PA, similar questions on motivators/facilitators, and barriers.

3. Information provision

- **Understanding and awareness**
  - explore what healthy eating means
  - know of any healthy eating/PA advice (source of advice)

- **Information about diet and lifestyle**
  - sources
  - views on information

- **Preferences**
  - what type of information
  - who should provide information (health professionals)
  - what format

For peer review only - http://bmjopen.bmj.com/site/about/guidelines.xhtml

4. Experience and views of dietary and lifestyle intervention

- **Participate in research study** - asked to change your **eating habits**

If YES

- where/how receive information
- types (delivery format)
- duration
- reasons for participating

***If NO,* why not? How to promote participation?**

- **Participate in research study** - asked to change your **physical activity**

If YES

- where/how receive information
- types (delivery format)
- duration
- reasons for participating

***If NO,* why not? How to promote participation?**

5. Preferences for intervention

- If we ask you to change your eating habits, for example cooked tomatoes, how would you feel about this? (soya products, fish, lycopene supplement)

If POSITIVE

- duration
- where/how receive information (leaflets, telephone)
- what would make it easier/harder
- when (after diagnosis/treatment)

***If NEGATIVE,* why? What would make it easier? Different type of intervention?**

- If we ask you to walk extra 30mins everyday, how would you feel about this?

If POSITIVE

- duration, type (brisk walking/stroll)
- where/how receive information (leaflets, telephone)
- what would make it easier/harder
- when (after diagnosis/treatment)

***If NEGATIVE,* why not? What would make it easier? Different type of intervention?**

For peer review only - http://bmjopen.bmj.com/site/about/guidelines.xhtml

Closing

- Thank you
- Any questions?
- Summary of results from the study?
- £15 voucher (thank you for your time)

*This topic guide was amended during data collection to include additional questions on concerns about body weight, familiarity with soya products and tomatoes as a feature of a traditional Caribbean diet. They were identified as salient topics in interviews with the first few participants.

For peer review only

For peer review only - http://bmjopen.bmj.com/site/about/guidelines.xhtml

Supplementary Material 2: Additional quotes from interviews

**Theme 1: Pre-cancer diet and lifestyle**	
Men who perceived themselves to have a healthy diet before prostate cancer did not change their diet	I don't, I don't make no changing. But as I says, I don't overdo things…I normally look for things what is fat free…That's why I, I eat, if I eat bread I eat brown bread. And if I drink tea with, tea with milk I will drink the green top. (Jamal)
The proposed dietary intervention-increasing tomato intake-was acceptable to men as they are commonly consumed as part of a traditional Caribbean diet	"Yeah, we cook with a lot of tomatoes. I cook it. That's part of what we call the seasonal, you know, tomato, onions and thyme, garlic…So tomato is in everything and the seasoning. (Thomas)
Men who perceived themselves to be physically active before prostate cancer did not think they should be more active, and advocated moderation	… I don't run marathons, (Laugh) you know, I never did that, you know…maybe I could do a bit more [walking], but I don't. And at the moment I don't think I would, you know. Because I consider myself, I do keep fairly active. (Jonah)
	Well I, I exercise at the allotment (Laugh) as much as possible…So we're active, you know, may- maybe not, no one is pushing you. What we, we do a little and I sit down, and don't push yourself… (Colton)
Pre-existing injuries or health conditions as a barrier to physical activity	"…I used to do karate for 20 something years but, er, I have an injury so I give it up" (Albert).
	Just walking, yes. Because, like, I don't want to do too much because of like, my asthma... (Shaun)
**Theme 2: Evidence: link between diet, lifestyle, and prostate cancer**	
Men did not think prostate cancer is linked to diet and lifestyle as they perceived themselves to have a healthy diet and lifestyle	I don't think it has anything to do with it. That's my view. I mean, you might differ. But I don't think it has anything to do with my diet. Because I think I eat all the right stuff. (Matthew)
Conflicting dietary messages	Er, I took cod liver capsule, I stopped because it's no good after the time, they say they find out it's no good for you, for, because, for long term they can give you prostate cancer too…Yeah. So if you listen, you know, [some of this diet] everything you do is no good, you know, so you eat nothing. (Albert)
Clinicians uncertain about the effectiveness of diet/lifestyle	he [doctor] can't give us any direction or any what to do at the moment, because they did say they're not sure… So they are not sure yet, so obviously

For peer review only

on delaying or preventing prostate cancer progression	they can't give you any advice. All they are trying is to keep fit, you know, eat healthy and things like that. (Dennis)
**Theme 3: Coping with prostate cancer: Just get on with it**	
Men who wanted to return to a 'normal' life after prostate cancer were less likely to change or seek dietary/lifestyle information	Well really, to be honest, I never accept, I knew the idea of prostate leads to cancer, but I never thought that I had got it, I had it. I never think, "Oh I've got cancer." Nothing like that, no…but I was lucky enough I, I took it at the early stage, so it didn't have to develop… Just carry on with my normal life, you know. Do everything in moderation. (Fabian)
Social network and support as a facilitator to dietary and physical activity changes	…She's [sister] telling me that, you know, sugar and dairy products, that's the other things I must stop eating them things. (Laugh)...Since she told me that, I normally use skimmed milk to make my tea and I make porridge… I just try and do what she said. Less sugar, less dairy products. I just try to eat less of those things. I don't know. (Joseph)
	And it's just being in a room, with other people, that are trying to do something. There's a positive energy about being in a room… it's always better when you- well, when you walk in a pack, with people that are motivated the same way that you are. (Thomas)
**Theme 4: Ageing: Those were the days, this is me now**	
Ageing as a barrier to strenuous physical activity; adapting to changes to the body	Yeah I still do a lot of activities. Decorator, painting and decorating. I can do all that, but it's only now I, the ladder, my body is saying to me, "Don't climb the ladder much anymore."…suddenly you find the aches and pains and I can't climb the ladder anymore. (Fabian)
	Age. Cricket I am, I think last I played cricket was about maybe 15, 20 years ago and you feel as you get older you're restricted. I can't see myself running here and running there to catch a bus and that's not good for the heart. (Errol)
Ageing heightened men's awareness of their health, especially in regards to their body weight	… You know, it's a wise thing to change your diet, and I think I am reaching a certain age, you know… I think I should sacrifice and get rid of those things [starchy foods] and try and keep myself on a balanced diet where I could then chop my weight and look after myself… (Dennis)

For peer review only

	….I want to go to the gym soon…they reckon I'm putting on a bit too much weight so go and take off some of the weight (Laugh). (Sebastian)
**Theme 5: Autonomy: It has to be something I enjoy**	
Men who made changes out of necessity-chronic health conditions- broke their diet as they felt restricted	…but after you're diabetic you say okay, that's not good that's not good so you leave that out… Yes, so, so what I don't eating, I try to follow the rules as much as I can, but rules are meant to be broken. (Laugh). If we, if you obey all the rules all the time you miss half the fun. (Laugh) (Errol)
Preference in regards to incorporating tomatoes into diet	I eat tomatoes, but I don't like cooked ones…Fresh ones, I buy loads of them and I just squash them, cut them up and just eat them in my, eat them in bread and thing with my dinner. (Joseph)
Views on a dairy-free diet	Cut out the skimmed milk from, from my cereal, but I like my cereal, every morning. That would be difficult. I have my cereal every morning. I could cut out the cheese, but the milk. That's a difficult one for me. (Matthew)
Have to like the food to eat it, regardless of its benefits	I don't like, I can't eat the English type food most most 'cause a lot of things I don't I never eat it. They say it's good to eat, and I never eat it… (Joseph)
	…but it must be, not all fruit is palatable to me. I, I'm, I'm must like it to eat it, whether it's good for me or not I must like it. (Errol)

For peer review only

COREQ (COnsolidated criteria for REporting Qualitative research) Checklist

A checklist of items that should be included in reports of qualitative research. You must report the page number in your manuscript where you consider each of the items listed in this checklist. If you have not included this information, either revise your manuscript accordingly before submitting or note N/A.

Topic	Item No.	Guide Questions/Description	Reported on Page No.
**Domain 1: Research team and reflexivity**			
Personal characteristics			
Interviewer/facilitator	1	Which author/s conducted the interview or focus group?	P8, L2
Credentials	2	What were the researcher's credentials? E.g. PhD, MD	P8, L7
Occupation	3	What was their occupation at the time of the study?	P8, L6
Gender	4	Was the researcher male or female?	P8, L6
Experience and training	5	What experience or training did the researcher have?	P8, L7-8
Relationship with participants			
Relationship established	6	Was a relationship established prior to study commencement?	No
Participant knowledge of the interviewer	7	What did the participants know about the researcher? e.g. personal goals, reasons for doing the research	P8, L6-7
Interviewer characteristics	8	What characteristics were reported about the inter viewer/facilitator? e.g. Bias, assumptions, reasons and interests in the research topic	P8, L8-12
**Domain 2: Study design**			
Theoretical framework			
Methodological orientation and Theory	9	What methodological orientation was stated to underpin the study? e.g. grounded theory, discourse analysis, ethnography, phenomenology, content analysis	P9, L4
Participant selection			
Sampling	10	How were participants selected? e.g. purposive, convenience, consecutive, snowball	P7, L11-17
Method of approach	11	How were participants approached? e.g. face-to-face, telephone, mail, email	P7, L9-11
Sample size	12	How many participants were in the study?	P7, L21
Non-participation	13	How many people refused to participate or dropped out? Reasons?	We did not rec
Setting			
Setting of data collection	14	Where was the data collected? e.g. home, clinic, workplace	P8, L2-4
Presence of non-participants	15	Was anyone else present besides the participants and researchers?	No
Description of sample	16	What are the important characteristics of the sample? e.g. demographic data, date	P9, L18-23; Tab
Data collection			
Interview guide	17	Were questions, prompts, guides provided by the authors? Was it pilot tested?	P8, L14-22; Sup
Repeat interviews	18	Were repeat inter views carried out? If yes, how many?	No
Audio/visual recording	19	Did the research use audio or visual recording to collect the data?	P8, L1-2
Field notes	20	Were field notes made during and/or after the inter view or focus group?	
Duration	21	What was the duration of the inter views or focus group?	P8, L
Data saturation	22	Was data saturation discussed?	P7, L20-22
Transcripts returned	23	Were transcripts returned to participants for comment and/or	No

For peer review only

Topic	Item No.	Guide Questions/Description	Reported on Page No.
		correction?	
**Domain 3: analysis and findings**			
Data analysis			
Number of data coders	24	How many data coders coded the data?	P9, L5-8
Description of the coding tree	25	Did authors provide a description of the coding tree?	N/A
Derivation of themes	26	Were themes identified in advance or derived from the data?	P9, L5-7
Software	27	What software, if applicable, was used to manage the data?	
Participant checking	28	Did participants provide feedback on the findings?	No
Reporting			
Quotations presented	29	Were participant quotations presented to illustrate the themes/findings? Was each quotation identified? e.g. participant number	Results, P10-18
Data and findings consistent	30	Was there consistency between the data presented and the findings?	P9, L5-12; L14-[illegible]
Clarity of major themes	31	Were major themes clearly presented in the findings?	Results, P10-18
Clarity of minor themes	32	Is there a description of diverse cases or discussion of minor themes?	Discussion, P1[illegible]

Developed from: Tong A, Sainsbury P, Craig J. Consolidated criteria for reporting qualitative research (COREQ): a 32-item checklist for interviews and focus groups. *International Journal for Quality in Health Care*. 2007. Volume 19, Number 6: pp. 349 – 357

Once you have completed this checklist, please save a copy and upload it as part of your submission. DO NOT include this checklist as part of the main manuscript document. It must be uploaded as a separate file.

For peer review only

Barriers and facilitators to healthy lifestyle and acceptability of a dietary and physical activity intervention among African Caribbean prostate cancer survivors in the UK: a qualitative study

Journal:	*BMJ Open*
Manuscript ID	bmjopen-2017-017217.R2
Article Type:	Research
Date Submitted by the Author:	09-Aug-2017
Complete List of Authors:	Er, Vanessa; University of Bristol School of Social and Community Medicine,<br>Lane, Athene; University of Bristol, School of Social and Community Medicine; NIHR Biomedical Research Unit in Nutrition, Diet and Lifestyle at University Hospitals Bristol NHS Foundation Trust and the University of Bristol<br>Martin, Richard; University of Bristol, Social Medicine; NIHR Biomedical Research Unit in Nutrition, Diet and Lifestyle at University Hospitals Bristol NHS Foundation Trust and the University of Bristol<br>Persad, Raj; Bristol Urological Institute, North Bristol NHS Trust, Southmead Hospital<br>Chinegwundoh, Frank; Barts Health NHS Trust, Department of Urology; City, University of London, School of Health Sciences<br>Njoku, Victoria; Barts Health NHS Trust, Department of Urology<br>Sutton, Eileen; University of Bristol School of Social and Community Medicine
**Primary Subject Heading**:	Qualitative research
Secondary Subject Heading:	Urology, Oncology, Nutrition and metabolism, Public health
Keywords:	Prostatic neoplasms, QUALITATIVE RESEARCH, diet, physical activity, Caribbean region, survivors

SCHOLARONE™
Manuscripts

1

Barriers and facilitators to healthy lifestyle and acceptability of a dietary and physical activity intervention among African Caribbean prostate cancer survivors in the UK: a qualitative study

Vanessa Er, J Athene Lane, Richard M Martin, Raj Persad, Frank Chinegwundoh, Victoria Njoku, Eileen Sutton

Corresponding author:

Dr Vanessa Er, School of Social and Community Medicine, University of Bristol, Canynge Hall, 39 Whatley Road, Clifton, Bristol, BS8 2PS, UK, E-mail: vanessa.er@bristol.ac.uk, Tel: +44 (0)117 928 7365.

Co-authors:

J Athene Lane, School of Social and Community Medicine, University of Bristol, 39 Whatley Road, Clifton, Bristol, BS8 2PS, UK. athene.lane@bristol.ac.uk

Richard M Martin, School of Social and Community Medicine, University of Bristol, 39 Whatley Road, Clifton, Bristol, BS8 2PS, UK. NIHR Bristol Nutrition Biomedical Research Unit. Level 3, University Hospitals Bristol Education & Research Centre, Upper Maudlin Street, Bristol, BS2 8AE, UK. MRC Integrative Epidemiology Unit, Oakfield House, Oakfield Grove, Bristol, BS8 2BN, UK. richard.martin@bristol.ac.uk

Raj Persad, Bristol Urological Institute, North Bristol NHS Trust, Southmead Hospital, Southmead Road, Bristol, BS10 5NB, UK. rajpersad@bristolurology.com

For peer review only

Frank Chinegwundoh, Department of Urology, Barts NHS Health Trust. The Royal London Hospital, Whitechapel Road, London E1 1BB, UK. School of Health Sciences, City, University of London. Northampton Square, London, EC1V 0HB, UK. frank.chinegwundoh@nhs.net

Victoria Njoku, Department of Urology, Barts NHS Health Trust. The Royal London Hospital, Whitechapel Road, London, E1 1BB, UK. victoria.njoku@bartshealth.nhs.uk

Eileen Sutton, School of Social and Community Medicine, University of Bristol, 39 Whatley Road, Clifton, Bristol, BS8 2PS, UK. e.sutton@bristol.ac.uk

Keywords

Qualitative research, prostatic neoplasm, diet, physical activity, Caribbean region, survivors

Word Count

5582 words

Abstract

Objectives: Diet and lifestyle may have a role in delaying prostate cancer progression, but little is known about the health behaviours of Black British prostate cancer survivors despite this group having a higher prostate cancer mortality rate than their White counterparts. We explored the barriers and facilitators to dietary and lifestyle changes and the acceptability of a diet and physical activity intervention in African Caribbean prostate cancer survivors.

For peer review only

Design: We conducted semi-structured in-depth interviews and used thematic analysis to code and group the data.

Participants and setting: We recruited 14 African Caribbean prostate cancer survivors via letter or at oncology follow-up appointments using purposive and convenience sampling.

Results: A prostate cancer diagnosis did not trigger dietary and lifestyle changes in most men. This lack of change was underpinned by five themes: pre-cancer diet and lifestyle, evidence, coping with prostate cancer, ageing, and autonomy. Men perceived their diet and lifestyle to be healthy and were uncertain about the therapeutic benefits of these factors on prostate cancer recurrence. They considered a lifestyle intervention as unnecessary because their prostate-specific-antigen (PSA) level was kept under control by the treatments they had received. They believed dietary and lifestyle changes should be self-initiated and motivated, but were willing to make additional changes if they were perceived to be beneficial to health. Nonetheless, some men cited advice from health professionals and social support in coping with prostate cancer as facilitators to positive dietary and lifestyle changes. A prostate cancer diagnosis and ageing also heightened men's awareness of their health, particularly in regards to their body weight.

Conclusions: A dietary and physical activity intervention framed as helping men to regain fitness and aid post-treatment recovery aimed at men with elevated PSA may be appealing and acceptable to African Caribbean prostate cancer survivors.

For peer review only

Strengths and limitations

- Our study is one of the first to explore the dietary and lifestyle practices and information seeking behaviour of African Caribbean men after a prostate cancer diagnosis in the UK.
- Views and preferences for a dietary and physical activity intervention were sought from men from diverse sociodemographic backgrounds and with different prostate cancer clinical history.
- We struggled to recruit men on active monitoring. It has been reported that Black African and Caribbean men in the UK were more likely to undergo radical treatment compared to their White counterparts, perhaps due to a tendency for their prostate cancers to be managed more aggressively by clinicians.
- We did not interview men's partners but it would be of interest to explore eating habits and food choices of the family with their partners to gain a deeper understanding of the roles and responsibilities regarding food within the household.
- This study reflects only intentions in response to a dietary and physical activity intervention which may differ from that in a real intervention.

For peer review only

Background

Prostate cancer is the second most common cancer in men worldwide, with over 1.1 million cases diagnosed in 2012.[1] In the UK, there are over 40,000 newly diagnosed prostate cancer cases and 10,000 deaths each year.[2] It affects Black men (Black African, Black Caribbean,

and Other Black) disproportionately, and in England, they are at twice the risk of being diagnosed with, and dying from prostate cancer than White men of the same age.[3] The African Caribbean community in the UK is established and make up a large proportion of older adults within the Black population,[4] hence they are more likely to be affected by prostate cancer as it is strongly related to age. Awareness of prostate cancer among Black men is low, with widespread misconceptions about the methods of diagnosis and treatment.[5] Their reluctance to be tested for prostate cancer has been attributed to a lack of communication and information from health professionals, mistrust of the health-care system, perceived threats to masculinity, fear of cancer and side effects of prostate cancer treatment.[5]

Radical treatments for prostate cancer are often accompanied by adverse effects that negatively impact on men's quality of life,[6] and the prostate-specific antigen (PSA) test cannot distinguish the majority of men with indolent cancer from the minority with aggressive, fatal cancer.[7] Since diet and lifestyle are thought to play a pivotal role in prostate carcinogenesis,[8] attention has turned towards the development of dietary and lifestyle interventions to prevent the progression of prostate cancer. A dietary and lifestyle intervention may appeal to African Caribbean prostate cancer survivors if it is congruent with the health beliefs of first generation African Caribbean, as they have been found to have a preference for natural treatment and place great emphasis on food for health maintenance.[9,10] These beliefs could be linked to their memories of growing up in the Caribbean, where fresh and natural foods are in abundance and the use of herbal remedies is widespread.[9,10]

In 2007, the World Cancer Research Fund and the American Institute for Cancer Research (WCRF/AICR), which published the most comprehensive and authoritative report on environmental exposures and cancer, concluded that there is some evidence that selenium and

For peer review only

foods rich in lycopene probably decrease the risk of prostate cancer while diets high in calcium increases prostate cancer risk.[8] This was followed by the WCRF/AICR continuous update project report in 2014, which found strong evidence for a link between body fatness and increased risk of advanced prostate cancer.[11] Physical activity has been linked to a lower risk[12,13] of prostate cancer progression and mortality, and has also been shown to alleviate the adverse effects of prostate cancer treatment.[14] However, current evidence suggests that prostate cancer survivors have sub-optimal diet and lifestyle profiles[15-18] and the evidence-base to support the development of successful and conclusive interventions is lacking and of poor quality.[19] There is a high prevalence of obesity among prostate cancer survivors,[15,17,18] and the proportion of prostate cancer survivors consuming '5 a day' of fruits and vegetables in most studies was relatively low, ranging from 15.6% to 30.7%.[15,16,18] Similarly, it has been reported that over 50% of prostate cancer survivors are inactive.[15-17] Whilst there is a body of literature on the health behaviours of prostate cancer survivors, these studies were conducted in predominantly White population and in the US, with limited qualitative studies exploring the facilitators of, and barriers to dietary and lifestyle changes.[20-24]

Dietary studies of African Caribbean in the UK (mainly first-generation) found that they have a lower energy and fat intake, and higher fruit and vegetable intake compared to the British population,[25] as they tend to adhere to a traditional African Caribbean diet that is rich in complex carbohydrates; including pulses, fruits and vegetables. The impact of dietary acculturation on second generation African Caribbean in the UK was evident, with this group more likely to adopt a Westernised diet which is high in fat and sugar, and low in fruits and vegetables.[26] To our knowledge, there is no qualitative study on the health behaviours of African Caribbean men after a prostate cancer diagnosis. It is important to understand the health behaviours and needs of African Caribbean prostate cancer survivors, so as to inform

For peer review only

the development of a dietary and lifestyle intervention which is both acceptable to this population and effective in improving prostate cancer outcomes. The aim of this qualitative study was to explore the facilitators and barriers to dietary and lifestyle changes, and the acceptability of a dietary and physical activity intervention among African Caribbean prostate cancer survivors to inform the development of a high-quality diet and physical activity randomised controlled trial.

Methods

Study population and ethical approval

We recruited men aged 18 and above who self-identified as African Caribbean, and had a clinically–confirmed prostate cancer diagnosis from two large cities in the UK by invitation letter through their consultants and in-person at oncology follow-up clinics. We used purposive sampling to select men across a range of ages, socioeconomic positions, marital status and treatment types to obtain a representative sample.[27] These factors have been identified in the literature to influence dietary and lifestyle changes after a prostate cancer diagnosis.[28] As a result of slow recruitment at the first site and time constraints – this study was conducted as part of a doctoral degree - we used convenience sampling[27] and focused our recruitment on another site with larger African Caribbean community. We aimed to recruit between 15 and 20 participants. The sample size was expected to obtain sufficient data to detect possible themes based on previous research[28,29] and advice from experienced qualitative researchers.[30-32] We were unable to recruit men on active monitoring. Nonetheless, no new themes were emerging from the data after 14 participants had been interviewed. Ethical approval was provided by the Greater Manchester West Research Ethics Committee (13/NW/0878).

For peer review only

In-depth interviews

All participants provided their written informed consent to take part in the study, for their interviews to be audio-recorded and quotes to be published. A researcher (VE) carried out in-depth, semi-structured interviews with participants at their homes in between 2014 and 2015. Interviews lasted between 45 minutes and 1 hour 40 minutes and participants received a £15 supermarket voucher at the end of the interview as a thank you for taking part. VE presented herself as a student who would like to know the participants' view on diet and prostate cancer as part of her studies. Although she has a Masters degree in nutrition, she did not reveal this to the participants. Her interest in researching diet and lifestyle of people from minority ethnic groups is due to her experience of growing up in Malaysia, a multiracial and multicultural country, and was surprised to discover that despite being disproportionately affected, there was a lack of research on the health behaviours of Black men in the UK diagnosed with prostate cancer.

VE used an interview topic guide (**Supplementary Material 1**) to direct discussions on men's dietary and lifestyle practices before and after a prostate cancer diagnosis, the reasons for dietary and/or lifestyle changes (or lack of), sources and views on dietary and lifestyle information, and views on a proposed dietary and physical activity intervention for prostate cancer survivors, which consists of increasing tomato intake, a dairy-free diet and 30 minute of brisk walking a day. The topic guide was informed by the literature, reviewed by a public and patient involvement group, and was refined twice based on preliminary findings from early interviews to include topics on body weight, familiarity with soya products, and tomatoes being a feature in the Caribbean diet.

Data analysis

For peer review only

The interviews and data analysis were carried out concurrently.[33] This was an iterative process such that salient issues or topics raised in early interviews could be explored or follow-up in subsequent interviews. All interviews were anonymised, transcribed verbatim and analysed using thematic analysis[34] with the aid of a qualitative data analysis computer software package (NVivo 10). The researcher (VE) used an inductive approach to code the data in 'chunks' to keep the context where the data originated, and developed an initial coding frame.[34] A portion of transcripts was coded independently by another researcher (ES) to ensure coding was consistent and was a balanced representation of participants' views. Throughout the coding process, extracts of data across the dataset were compared to check for similarities and differences.[34] The coding framework was refined and revised by VE and ES as the analysis progressed. We identified, grouped and conceptualised patterns within and across the dataset which are salient to the participants into themes.[34] Field notes and analytic memos written by VE were used to explore how codes and themes related to each other and aided the conceptualisation of key themes. VE and ES discussed any discrepancies and finalised the themes. All names presented here refer to pseudonyms.

Results

The characteristics of the participants are presented in **Table 1**. At the time of interview, the age of the participants ranged from 52 to 80 years, with a median age of 71.5 years. Most of the men were married or living with a partner; diagnosed with prostate cancer less than five years at the time of the interview; and had radiotherapy and adjuvant hormone therapy as treatment. With the exception of five men, the rest of the participants were retired. Most men have or previously had semi-routine or routine occupation.

For peer review only

10

Table 1 Characteristics of participants

Pseudonym	Age	Marital status	Years since diagnosis	Cancer stage	Treatment	Co-morbidities
Aaron	79	M/P	≤1	Localised	Not started[a]	Hypertension, Gastro-oesophageal reflux disease
Dennis	56	M/P	≤1	Localised	Not started[a]	Hypertension
Jonah	75	M/P	>10	Locally advanced	RT/HT	-
Fabian	73	M/P	>1 and <5	Locally advanced	RT/HT	Type 2 Diabetes
Colton	77	M/P	>10	Locally advanced	RT/HT	Hyperthyroidism
Albert	65	M/P	≥5 & <10	Localised	RT/HT	Hay fever
Jamal	70	S/W	>1 and <5	Locally advanced	RT/HT	-
Matthew	80	S/W	>10	Locally advanced	RT/HT	Type 2 Diabetes
Irvin	79	S/W	>1 and <5	Localised	RT/HT	-
Errol	76	S/W	≤1	Locally advanced	RT/HT	Type 2 Diabetes
Shaun	68	M/P	≤1	Localised	RT/HT	Type 2 Diabetes, Asthma
Joseph	63	M/P	≤1	Localised	Prostatectomy	-
Sebastian	61	M/P	≤1	Localised	Prostatectomy	-
Thomas	52	M/P	≤1	Localised	Prostatectomy	-

RT/HT: radiotherapy and adjuvant hormone therapy. M/P: Married or living with a partner. S/W: Single or widowed.
[a] Had not started treatment at the time of interview.

Five themes were identified from the analysis which captured the barriers and facilitators to dietary and lifestyle changes, the rationale for seeking (or not) dietary and lifestyle information, and men's views on the target population, timing, and acceptability of a proposed dietary and physical activity intervention. Additional quotes relating to each theme are provided in **Supplementary Material 2.**

Theme 1: Pre-cancer diet and lifestyle

w only

Most men who perceived their diet to be healthy before a prostate cancer diagnosis ate as usual. Some men had already made positive dietary changes prompted by other health problems such as diabetes, while others questioned what changes could be made if they were already eating healthily.

> *Well, but erm - change to what? (Laugh) What do you change to, in those circumstances? If you eat, you know, if you eat healthy food, meat, vegetables, salads, fruits, what do you change to? (Jonah)*

Thus, most of the men did not seek further dietary information after a prostate cancer diagnosis as they perceived themselves as having a healthy lifestyle and knowing what constitutes a healthy diet. When men were asked for their views on a dietary intervention which would require them to eat more tomatoes and cut out dairy products, they were amenable to it because it complemented their existing diet. They explained that tomatoes are commonly consumed as part of a traditional Caribbean diet, while dairy products only account for a small fraction of it. Similarly, a proposed physical activity intervention which involves brisk walking for 30 minutes a day was considered achievable, with several men already doing it in their daily lives.

Men who perceived themselves to be active before prostate cancer also did not think they needed to increase their physical activity. For men, being active meant being up on their feet and 'doing things', but they advocated moderation. As one participant (Errol) noted: *"I do the exercise in my work. 'Cause when you're a builder, you don't sit, you don't do nothing, you have to move around all the time, you know. So that to me enough exercise."* A few men wanted to be more active but they were impeded by health conditions or sports injuries. Conversely, men who made changes acknowledged that their diet and lifestyle before

For peer review only

prostate cancer could have been better and a prostate cancer diagnosis acted as a catalyst for the change.

…But since I was diagnosed, er, we just, I would say about, the beef probably come out, we cut down beef a bit, you know? … they always say too much red beef is not good for you and that. So we just cut it down, you know? (Albert)

Theme 2: Evidence: link between diet, lifestyle, and prostate cancer

Men's views on the causes of cancer varied, ranging from genetic factors, fate or chance, stress (not enough rest), diet and lifestyle to food production (chemicals). However, the majority of men were aware that Black men have higher risk of prostate cancer, and some described it as an 'old man's complaint'. A few men cited a lack of evidence that prostate cancer is linked to diet and lifestyle to support their view. This was contrasted with men's belief that smoking increases the risk of cancer due to widely established evidence that it causes lung cancer.

…up until now they haven't come out with err, err, anything positive to say, "This is the result, this is the main reason why black men from the Caribbean or West Africa have a, how I say, the most higher rate."…So I don't think that I am in any position to think of, because of our diet or because of what we eat, do you understand? (Dennis)

Men who believed that prostate cancer is not connected to diet were less likely to seek dietary information. This was partly due to their mistrust of the dietary messages from media which they regarded as conflicting. They preferred receiving information from health professionals who they regarded as experts and a trusted source of health information, and that had a positive influence on their health behaviour. For example, one man (Colton) started eating tomatoes and drinking pomegranate juice because his clinicians informed him that it could be

For peer review only

beneficial for prostate cancer: *"she [dietician] said with, with the prostate cancer that I've got I should eat a lot of tomato. Tomato is good for the prostate cancer. My doctor before, Doctor M, told me that if I drink pomegranate juice it's a little bit helpful as well…That's what I buy, we buy, things, we buy pomegranate juice."* However, only a few men reported that they received dietary or lifestyle advice from health professionals, with some explaining that their doctor was uncertain about the effectiveness of diet and lifestyle on prostate cancer.

There was however a small number of men who believed that diet and lifestyle are important for cancer prevention, but they did not think diet and lifestyle have any impact on cancer progression, particularly if their treatment was effective: *"So, so what the difference that it make to, it's not going to affect me now because I'm, I've [passed that stage] with the prostate cancer" (Albert)*. Consequently, men suggested that a dietary and physical activity intervention should be offered before treatment, as the effect of the intervention would not be confounded by the treatment, and if the intervention works, treatment would not be necessary. The significance of PSA became apparent when men explained their views, as a high or rising PSA level after treatment was perceived as indicative of recurrent prostate cancer.

> *Well, do it before treatment 'cause it [PSA level] might move and it might not. 'Cause if it moves then obviously I've got to go in for treatment. And if it doesn't move, obviously the longer it stays away then the better it is for me. And I wouldn't actually need the radiotherapy. (Sebastian)*

Accordingly, they believed that interventions should be targeted at men who have a high PSA level and were more likely to adopt dietary or lifestyle advice if it was shown to reduce or keep PSA level low.

For peer review only

Theme 3: Coping with prostate cancer: just get on with it

Men had differing approaches to coping with prostate cancer which influenced whether they made positive changes to their diet and lifestyle. Those who were sanguine about prostate cancer tended to 'take it as it comes' and thus less likely to change their diet and lifestyle and were not interested in gaining further dietary and lifestyle information. Similarly, men who considered their cancer as treated just wanted to move on and not dwell upon it further.

> *Well me don't really want to get no more information, because so far I'm not living with the thing again. So I don't really want to get no more information, I just try to do, the, my best, try to help myself in many ways. (Jamal)*

These approaches were directly contrasted with those of men who were actively seeking ways to alleviate the side effects they experienced and wanting to be fit again. Thomas spoke about exercise as a way of making himself feel better as it felt like he was doing something for his body, rather than relying on medication for depression or pain: "*If there's something that you need, to motivate yourself and exercise, might trigger it off. I'm not sure, but I know that sitting down, taking tablets, isn't going to do it, or not even diet, to that point.*"

It became evident that social networks and support were important for men in coping with prostate cancer, and facilitated positive dietary and lifestyle changes. Partners, family and friends acted as a 'change agent' to enable these changes. The role of partner or wife in initiating dietary changes and encouraging men to live healthier is highlighted in this comment.

> *… more of less it's the wife that is leading the process [dietary change] really. (Laugh) Yeah, she's the one doing the shopping, she's the one providing the food. And now*

For peer review only - http://bmjopen.bmj.com/site/about/guidelines.xhtml

she's, she's going and, and do all the shopping and buy whatever she thinks that's good for our health. (Dennis)

Several men commented on the importance of social support to stay physically active. They were very positive about group exercise citing shared purpose and motivation as an attraction to them, as one participant (Matthew) commented: *"what group exercises do is to encourage you and encourage others to follow. That is why I used to like going to the gym, 'cause you had people there who were there for the same purpose."*

Whether men made dietary and lifestyle changes was also dependent on their priorities and concerns after cancer treatment. Men typically experienced side effects from treatments including incontinence and erectile dysfunction. Thus, coping and adapting to these side effects were their main priorities. Primarily, incontinence was reported as a barrier to physical activity.

I want to go to the gym soon, but like I said, because I'm so wet I won't have – I'm hoping that it will ease up a little bit so I can get to do something else….(Sebastian)

Loss of income because of a prostate cancer diagnosis could also have a knock-on effect on opportunities for physical activity. Sebastian who was self-employed had financial worries and his main priority was to return to work, for without an income, he could not afford to pay for gym membership.

Theme 4: Ageing: those were the days, this is me now

Most men used to play sports and acknowledged the health benefits of physical activity, but old age was the most commonly cited reason for not doing strenuous physical activity. For

For peer review only

men who had retired, life after work was portrayed as a time to rest (slow down) and for leisure. Most men (aged 70 and above) viewed themselves as too old to be playing sports and gentle exercise as safer and more appropriate for their age. Therefore, a brisk walking intervention was perceived as safe and acceptable by men in the study.

> *I'm too old to go to the gym man. I don't want to go to the gym to get a heart attack. (Laughter) Because I don't know, I don't go there [to lift up] these big great things, I go…on the treadmill. (Laugh) I used to go on it but I don't want to do all these type of things now. (Jamal)*

Men made frequent references to their body when describing their physical limitations. They also talked about having to accept and adapt to the changes in their body as they aged. As one participant (Joseph) noted: *"Age is against me now (laugh). I don't feel much pain, but you can tell on the body. Your body can't cope with that. I know it can't cope with it anymore."*

Younger men (under 65 years old) however had no qualms about performing higher intensity physical activity. For Thomas, being back in the gym and having the ability to do the exercises just as before, was a sign of recovery - "*erm- because I've been in a gym before, it's going back to an old friend, and actually familiarising yourself with it, and being told- and telling yourself that you- you're back on the road to recovery…To be back in that gym environment again, it- it was lovely…*" Similarly, Dennis who had a prostate biopsy before the interview hoped to continue playing cricket for his club - *"Well, I used to play every Sunday…but since I had the operation I haven't played…when I am feeling healthy and okay I will"*.

For peer review only

On the other hand, men had more awareness of their health as they aged because they perceived that they were more susceptible to illness, so having a healthy diet and lifestyle became more relevant to them. For the men in this study, ageing also shifted the focus from providing for the family to taking care of themselves. There was also a perception espoused by men that getting older made one wiser and thus more sensible. Some men noticed that they put on weight more easily as they aged, mainly because they were less active than before. That prompted men to eat less, cut down food portion sizes, alcohol intake or exercise more.

> *Yeah, that's why I said don't want to eat fatty things. I don't want to be bloated out, put it that way…Yeah, yeah, I don't want to get big, fat and clumsy man, where I can't move about and things like that. So I, I watch, I know what I'm eating. (Jamal)*

The younger men who experienced side effects from prostate cancer treatments, especially incontinence and fatigue, and thus unable to perform strenuous physical activity also expressed concerns about their weight and wanted to regain fitness to cope with these side effects.

> *… she [trainer] said, "You've got a bigger belly than you had before, and if you want- what do you want to get out of this?" And I said, "I'd like to probably get my shape back, and just lose a little bit of weight…" (Thomas)*

Theme 5: Autonomy: it has to be something I enjoy

Overall, men believed that an individual has to be personally motivated to make dietary and lifestyle changes so that they would be maintained long-term. Men who made changes to their diet and lifestyle out of necessity due to co-morbidities such as diabetes, felt restricted and thus sometimes broke the rules. Although the proposed dietary intervention which

For peer review only

required men to increase intake of tomatoes was acceptable for most men, they expressed different preferences in regards to taste and ways of incorporating tomatoes in their diet. A few men favoured whole tomatoes over lycopene supplement (tablets) because they were suspicious of its content and production process.

> *It would be easier, but- but, I mean, it's- yeah, it would be easier, without a doubt. If you could take a tomato tablet that does it, but then it's what goes into making the tablet. With a tomato, a fresh tomato, you know what you're getting, don't you? (Thomas)*

Similarly, men in general were amenable to a dairy-free diet but they had reservations about substituting dairy products with soya, because they were unfamiliar with soya products and perceived dairy substitutes as unnecessary due to a low dairy intake. Nevertheless, there were a few men who were hesitant to give up milk which they usually consumed with breakfast cereals or in porridge. Some men also commented that they have to enjoy or like the food to eat it, regardless of its health benefits. One man rejected the idea of eating tomatoes every day despite being told of the potential protective effect of tomatoes from prostate cancer – *"No I won't eat all that…normally I use one for the salad - if I make salad – [I wouldn't want to do]… You know? I don't eat every day…I don't feel like doing it, and I don't want to do it. (Laugh)"* (Irvin).

Finally, the majority of the men interviewed preferred to walk on their own instead of taking part in a group exercise, even though they recognised the support and motivation that it could provide. Men explained that each person has his own schedule and routine, thus it is easier to walk on their own and at a pace that suited them.

For peer review only

Na, because again I have to, they want, when I'm ready to do it they won't be ready, that's the problem. That's why I have to do my own. (Albert)

Discussion

Most men in our study did not change their diet and lifestyle after a prostate cancer diagnosis. The lack of change was underpinned by five key themes: pre-cancer diet and lifestyle, evidence, coping with prostate cancer, ageing, and autonomy. The implications for research and practice are described below.

Design of dietary and lifestyle intervention for prostate cancer survivors

The proposed dietary and physical activity intervention was acceptable to men in this study, but they suggested it should be aimed at men with an elevated or rising PSA level, including those who are on active monitoring. The majority of dietary and lifestyle interventions for cancer survivors have been conducted after treatment with the aim of improving general health, and quality of life, but this finding suggests that men may be more interested in the clinical effect of interventions. Consequently, men perceived the proposed intervention as unnecessary because their PSA level was kept under control by the treatments they had received, which supports the findings of a previous study[28] and highlights the significance of using PSA as an outcome measure, despite criticism of its usefulness as a surrogate end-point in prostate cancer trials.[35,36] It also illustrates the challenges of informing men that dietary or lifestyle factors may act on prostate cancer independently of PSA.

This study also showed that men valued autonomy in their food and lifestyle choices and they might change their diet and lifestyle if it was deemed necessary and beneficial for health. Similar studies examining healthy eating using gender theory found that resistance among

For peer review only

men was linked to masculine ideals of autonomy and rationality.[37] The authors suggest that healthy eating initiatives and materials could be tailored to men by emphasising personal choices and responsibility.[37] Most men in our study preferred individual over group exercise, which differed from previous studies reporting that men enjoyed the opportunity to socialise when exercising.[24,38] Nonetheless, men were willing to participate in weekly group exercise provided that a time and place that is convenient for all could be found. Therefore, either a choice of home-based intervention only or in combination with weekly supervised group exercise may appeal to African Caribbean prostate cancer survivors.

Provision of dietary and lifestyle advice and support

Evidence from existing literature suggests that most men were uncertain about the role of diet and lifestyle in preventing prostate cancer progression or recurrence, but a strong belief in cancer-diet relationship was a predictor of dietary changes.[20-22,28,29] As with previous studies,[20,23,24,28,29] our findings suggest that advice from health professionals can strengthen men's beliefs on the relationship of prostate cancer with diet and lifestyle, influencing men to make positive lifestyle changes. Most men in our study did not recall receiving dietary or lifestyle information from health professionals, perhaps due to their hesitance on providing dietary and lifestyle advice because of a lack of evidence on diet and prostate cancer and awareness of relevant guidelines for cancer survivors.[28,39] Nonetheless, it is becoming clear that obesity is associated with higher risk of advanced and fatal prostate cancer,[11] and health professionals could potentially play a role in influencing men's health behaviour.

Awareness of supporting services was low among our participants. The UK National Cancer Survivorship Initiative (NCSI) recommend a 'recovery package' which consists of a combination of interventions, including a patient education and support event (health and

For peer review only

wellbeing clinic) to be offered to all cancer survivors.[40] A report by the London Cancer Alliance found that there was no standardised definition or structure for health and wellbeing clinics in their partner NHS trust providers.[41] Therefore, more research is needed in designing and evaluating the referral pathways for healthy lifestyle services, and developing a coherent model for health and wellbeing clinics.

Active ageing and body weight

In line with previous research,[42,43] misconceptions about the harms or risks of strenuous physical activity in old age such as heart attack and feeling breathless were shared by men in this study. Older men also felt that going to the gym was incompatible with their age as for them it was typically associated with young people 'pumping iron'. Although there has been much effort in the UK and developed countries to promote active ageing, this shows that further work is needed to challenge prevailing social norms on ageing and physical activity to ensure men stay active as they age. Older African Caribbean prostate cancer survivors should be informed that resistance exercise is beneficial to their health especially for preventing loss of muscle mass in men on androgen deprivation therapy.

Ageing or being diagnosed with prostate cancer also heightened men's awareness of their health and prompted several men to make positive changes to their diet and lifestyle for maintenance of general health. Concerns about body weight among older men and prostate cancer survivors have been reported in several studies.[21,28,37] In this study, men wanted to maintain a healthy weight to avoid having a 'big belly'. This differs from previous studies which found that men lose weight for health purposes rather than for appearance (body image).[44,45] Men in this study watched their weight by eating smaller portion sizes, having light meals (less starchy food) and increasing their physical activity, contradicting previous

For peer review only

studies which found that men preferred to lose weight through exercising because it is regarded as a masculine pursuit,[44] while 'dieting' practices such as increasing fruits and vegetable intake and reducing food portion sizes have been perceived to be feminine.[45,46]

Younger men also expressed interest in losing weight and regaining fitness after a period of inactivity due to side-effects of treatment. Similar to previous studies, men who experienced urinary incontinence were unsure about the type and intensity of physical activity that they could perform for fear of exacerbating their condition.[23,24,38] Thus, a supervised physical activity intervention would appeal to men to help them to lose weight and alleviate the side effects of treatments. A physical activity intervention could also be framed as a way for men to take charge of their recovery for better health.

Our study is one of the first to explore the dietary and lifestyle practices and information seeking behaviour of African Caribbean men after a prostate cancer diagnosis in the UK. Additionally, views and preferences for a dietary and physical activity intervention were sought from men to inform the design of a tailored intervention. It included men from diverse sociodemographic backgrounds and with different prostate cancer clinical history.

We struggled to recruit men on active monitoring. It has been reported that Black African and Caribbean men in the UK were more likely to undergo radical treatment compared to their White counterparts,[47] perhaps due to a tendency for their prostate cancers to be managed more aggressively by clinicians.[48] Additionally, the proportion of men on active monitoring is relatively low – 12% in UK in 2009[48] - compared to other prostate cancer treatments. We did not interview men's partners. It would be of interest to explore eating habits and food choices of the family with their partners to gain a deeper understanding of the roles and

For peer review only

responsibilities regarding food within the household, and contrast with the diet of single/widowed men who live alone. There was an indication that men preferred to consume tomatoes over a lycopene supplement, which requires further investigation. Furthermore, questions on format of delivery (telephone-based, print-based, supervised physical activity) and ways of monitoring adherence (food diary, pedometers) were not explored in all interviews, precluding comparisons between men. In future research, clear and simple visual aids could be used to present and facilitate discussions on the proposed intervention. Finally, this study reflects only intentions in response to a dietary and physical activity intervention; this may differ from that in a real intervention.

Conclusions

A prostate cancer diagnosis did not trigger dietary and lifestyle changes in this group of men who mostly perceived their diet and lifestyle to be healthy and were uncertain about the therapeutic benefits of diet and lifestyle on prostate cancer recurrence. They considered an intervention as unnecessary because their PSA level was kept under control by the treatments they had received. Men believed dietary and lifestyle changes should be self-initiated and motivated but were willing to make additional changes if they were perceived to be beneficial to health. Nonetheless, some men cited advice from health professionals and social support in coping with prostate cancer as facilitators to positive dietary and lifestyle changes. Furthermore, a prostate cancer diagnosis and ageing heightened men's awareness of their health, particularly in regards to their body weight. Therefore, a dietary and physical activity intervention which enhances men's autonomy, framed as helping men to regain fitness and aid post-treatment recovery, and is aimed at men with elevated PSA may be appealing and acceptable to African Caribbean prostate cancer survivors.

For peer review only

Declarations

Contributing statement

VE, ES, JAL, and RMM conceived the study; VE, ES, FC, RJ and VN contributed to the design and conduct of the research, and acquisition of data; VE and ES analysed the data; VE, ES, RMM and JAL wrote the first draft of the paper; ES, JAL, and RMM provided supervision; VE, ES, JAL, and RMM have primarily responsibility for final content. All authors were involved in revising the paper, and read and approved the final version of the paper.

Acknowledgement

The authors would like to thank all participants for sharing their views and experience; Dr. Mona Jeffreys and the Bristol Nutrition BRU prostate patient and public involvement group for their input into the study design and documents; and all staff who had helped with the recruitment of participants, especially Dr. Karen Tipples.

Disclaimers

The views expressed herein are those of the authors and do not necessarily reflect those of Cancer Research UK, the NHS, the NIHR, or the Department of Health.

Competing interest

All authors have completed the ICMJE uniform disclosure form at www.icmje.org/coi_disclosure.pdf and declare: no support from any organisation for the submitted work; no financial relationships with any organisations that might have an interest in the submitted work in the previous three years; no other relationships or activities that could appear to have influenced the submitted work.

For peer review only

Funding

This work was supported by the National Institute for Health Research (NIHR) Bristol Nutrition Biomedical Research Unit. The unit is a partnership between University Hospitals Bristol NHS Foundation Trust and the University of Bristol. VE was a recipient of a 3-year PhD studentship at the Bristol Nutrition Biomedical Research Unit from 2012-2015. RMM and JAL are recipients of CRUK programme funding (C18281/A19169).

Data sharing statement

The datasets generated and/or analysed during the current study are not publicly available as participants may be identifiable, but are available from the corresponding author on reasonable request.

Ethics approval and consent to participate

Ethical approval was provided by the Greater Manchester West Research Ethics Committee (13/NW/0878). All participants provided their written informed consent to take part in the study.

For peer review only

References

1. Ferlay J, Soerjomataram I, Dikshit R, et al. Cancer incidence and mortality worldwide: sources, methods and major patterns in GLOBOCAN 2012. *Int J Cancer* 2015;136(5):E359-86
2. Office for National Statistics. Cancer incidence and mortality in the United Kingdom 2008-2010 http://webarchive.nationalarchives.gov.uk/20151013220531/http://www.ons.gov.uk/ons/rel/cancer-unit/cancer-incidence-and-mortality/2008-2010/index.html2012 [accessed July 2017].
3. Lloyd T, Hounsome L, Mehay A, et al. Lifetime risk of being diagnosed with, or dying from, prostate cancer by major ethnic group in England 2008-2010. *BMC Med* 2015;13:171.
4. Centre for Policy on Ageing (UK). The ageing of the ethnic minority populations of England and Wales: findings from the 2011 census. UK, 2013.
5. Pedersen VH, Armes J, Ream E. Perceptions of prostate cancer in Black African and Black Caribbean men: a systematic review of the literature. *Psychooncology* 2012;21(5):457-68.
6. Bangma CH, Roemeling S, Schroder FH. Overdiagnosis and overtreatment of early detected prostate cancer. *World J Urol* 2007;25(1):3-9.
7. Lilja H, Ulmert D, Vickers AJ. Prostate-specific antigen and prostate cancer: prediction, detection and monitoring. *Nat Rev Cancer* 2008;8(4):268-78.
8. World Cancer Research Fund, American Institute for Cancer Research. Food, nutrition, physical activity, and the prevention of cancer: a global perspective. Washington DC: AICR 2007.
9. Brown K, Avis M, Hubbard M. Health beliefs of African-Caribbean people with type 2 diabetes: a qualitative study. *Br J Gen Pract* 2007;57(539):461-69.
10. Nanton V, Dale J. 'It don't make sense to worry too much': the experience of prostate cancer in African-Caribbean men in the UK. *Eur J Cancer Care* 2011;20(1):62-71.

American Institute for Cancer Research. Diet, nutrition, physical activity and prostate cancer. Continuous update project: World Cancer Research Fund, 2014.

12. Kenfield SA, Stampfer MJ, Giovannucci E, et al. Physical activity and survival after prostate cancer diagnosis in the health professionals follow-up study. *J Clin Oncol* 2011;29(6):726-32.
13. Bonn SE, Sjolander A, Lagerros YT, et al. Physical activity and survival among men diagnosed with prostate cancer. *Cancer Epidemiol Biomarkers Prev* 2015;24(1):57-64.
14. Gardner JR, Livingston PM, Fraser SF. Effects of exercise on treatment-related adverse effects for patients with prostate cancer receiving androgen-deprivation therapy: a systematic review. *J Clin Oncol* 2014;32(4):335-46.
15. LeMasters TJ, Madhavan SS, Sambamoorthi U, et al. Health behaviors among breast, prostate, and colorectal cancer survivors: a US population-based case-control study, with comparisons by cancer type and gender. *J Cancer Surviv* 2014;8(3):336-48.
16. Blanchard CM, Courneya KS, Stein K. Cancer survivors' adherence to lifestyle behavior recommendations and associations with health-related quality of life: Results from the American Cancer Society's SCS-II. *J Clin Oncol* 2008;26(13):2198-204.
17. Coups EJ, Ostroff JS. A population-based estimate of the prevalence of behavioral risk factors among adult cancer survivors and noncancer controls. *Prev Med* 2005;40(6):702-11.

For peer review only

18. Rogers LQ, Courneya KS, Paragi-Gururaja R, et al. Lifestyle behaviors, obesity, and perceived health among men with and without a diagnosis of prostate cancer: A population-based, cross-sectional study. *BMC Public Health* 2008;8:43-58.
19. Hackshaw-McGeagh LE, Perry RE, Leach VA, et al. A systematic review of dietary, nutritional, and physical activity interventions for the prevention of prostate cancer progression and mortality. *Cancer Causes Control* 2015;26(11):1521-50.
20. Satia JA, Walsh JF, Pruthi RS. Health behavior changes in White and African American prostate cancer survivors. *Cancer Nurs* 2009;32(2):107-17.
21. Coa KI, Smith KC, Klassen AC, et al. Exploring important influences on the healthfulness of prostate cancer survivors' diets. *Qual Health Res* 2015;25(6):857-70.
22. Mroz LW, Chapman GE, Oliffe JL, et al. Prostate cancer, masculinity and food. Rationales for perceived diet change. *Appetite* 2010;55(3):398-406.
23. Craike MJ, Livingston PM, Botti M. An exploratory study of the factors that influence physical activity for prostate cancer survivors. *Support Care Cancer* 2011;19(7):1019-28.
24. Keogh JW, Patel A, MacLeod RD, et al. Perceived barriers and facilitators to physical activity in men with prostate cancer: possible influence of androgen deprivation therapy. *Eur J Cancer Care (Engl)* 2014;23(2):263-73.
25. Vyas A, Greenhalgh A, Cade J, et al. Nutrient intakes of an adult Pakistani, European and African-Caribbean community in inner city Britain. *J Hum Nutr Diet* 2003;16(5):327-37.
26. Sharma S, Cade J, Riste L, et al. Nutrient intake trends among African-Caribbeans in Britain: a migrant population and its second generation. *Public Health Nutr* 1999;2(4):469-76.
27. Ritchie J, Lewis J, Nicholls CM, et al. Qualitative research practice: A guide for social science students and researchers. London: Sage 2013.
28. Avery KN, Donovan JL, Horwood J, et al. The importance of dietary change for men diagnosed with and at risk of prostate cancer: a multi-centre interview study with men, their partners and health professionals. *BMC Fam Pract* 2014;15:81.
29. Horwood JP, Avery KN, Metcalfe C, et al. Men's knowledge and attitudes towards dietary prevention of a prostate cancer diagnosis: a qualitative study. *BMC Cancer* 2014;14:812.
30. Baker SE, Edwards R, Doidge M. How many qualitative interviews is enough?: Expert voices and early career reflections on sampling and cases in qualitative research. 2012
31. Guest G, Bunce A, Johnson L. How many interviews are enough? An experiment with data saturation and variability. *Field methods* 2006;18(1):59-82.
32. Hennink MM, Kaiser BN, Marconi VC. Code saturation versus meaning saturation: How many interviews are enough? *Qual Health Res* 2017;27(4):591-608.
33. Glaser B, Strauss, A. The discovery of grounded theory: strategies for qualitative research. New York: Aldine 1967.
34. Braun V, Clarke V. Using thematic analysis in psychology. *Qualitative Research in Psychology* 2006;3(2):77-101.
35. Thompson IM. PSA: a biomarker for disease. A biomarker for clinical trials. How useful is it? *J Nutr* 2006;136(10):2704S.
36. Collette L. Prostate-specific antigen (PSA) as a surrogate end point for survival in prostate cancer clinical trials. *Eur Urol* 2008;53(1):6-9.
37. Gough B, Conner MT. Barriers to healthy eating amongst men: a qualitative analysis. *Soc Sci Med* 2006;62(2):387-95.

For peer review only

38. Bourke L, Doll H, Crank H, et al. Lifestyle intervention in men with advanced prostate cancer receiving androgen suppression therapy: A feasibility study. *Cancer Epidemiol Biomarkers Prev* 2011;20(4):647-57.
39. Sutton E, Hackshaw-McGeagh LE, Aning J, et al. The provision of dietary and physical activity advice for men diagnosed with prostate cancer: a qualitative study of the experiences and views of health care professionals, patients and partners. *Cancer Causes Control* 2017;28(4):319-329.
40. Department of Health (England), Macmillan Cancer Support, NHS Improvement (England). Living with & beyond cancer: taking action to improve outcomes. London, UK, 2013.
41. London Cancer Alliance. Health and wellbeing events mapping. UK, 2015.
42. Grant BC. 'You're never too old': beliefs about physical activity and playing sport in later life. *Ageing and Society* 2001;21:777-98.
43. Cousins SOB. Exercise, aging, and health: Overcoming barriers to an active old age. Philadelphia, PA: Taylor & Francis 1998.
44. De Souza P, Ciclitira KE. Men and dieting: A qualitative analysis. *J Health Psychol* 2005;10(6):793-804.
45. Gough B. 'Real men don't diet': an analysis of contemporary newspaper representations of men, food and health. *Soc Sci Med* 2007;64(2):326-37.
46. Jensen KO, Holm L. Preferences, quantities and concerns: socio-cultural perspectives on the gendered consumption of foods. *Eur J Clin Nutr* 1999;53(5):351-59.
47. Evans S, Metcalfe C, Patel B, et al. Clinical presentation and initial management of Black men and White men with prostate cancer in the United Kingdom: the PROCESS cohort study. *Br J Cancer* 2010;102(2):249-54.
48. Hounsome L, Iles M, Verne J. Treatment routes in prostate cancer. UK: The National Cancer Intelligence Network, 2012.

For peer review only

Supplementary Material 1

Diet and Lifestyle after Prostate Cancer Diagnosis

Topic Guide

Aims and Objectives

The aim of this study is to gather the evidence for dietary and lifestyle changes in prostate cancer survivors to inform a dietary and lifestyle intervention for randomised trials. This will involve exploring:

- Motivators and barriers to changes in dietary and lifestyle behaviours
- Sources of information on diet and lifestyle
- Experience and views of dietary and lifestyle interventions
- Preferences for intervention (delivery format)

Introduction

- Thank you for participating
- Introduce self and Uni Bristol
- Introduce the study: what is it about and why (limited information)
- Talk through key points:
  - length of interview
  - interview like a discussion, but will cover key topics
  - no right or wrong answers
  - participation is voluntary, rights to withdraw
  - recording interview (concentrate on what you are saying, accuracy)
- Confidentiality and anonymity, how findings will be reported
- Thank you payment
- Questions?
- Happy to proceed? Sign consent form

START RECORDING

For peer review only

For peer review only - http://bmjopen.bmj.com/site/about/guidelines.xhtml

University of BRISTOL

University Hospitals Bristol NHS
NHS Foundation Trust

1. Background and medical history

- **Age; household** (live alone or with others)
  - relationship with
  - married/widowed/single/partner

- **Place of birth; religion**
  - if born outside of UK, where? how long living in the UK
  - follow any religion

- Main **daytime activity**
  - working or not: details

- **Other interests/activities**

- **Prostate cancer**
  - when diagnosed
  - treatment(s)

- **Overall health**
  - health conditions that **may affect diet/lifestyle** e.g. diabetes, heart problems, high blood pressure

- Importance and practicalities attached to **food and physical activity**
  - family/culture (who does the cooking/food purchase)
  - involvement in sports/food-related organisation (e.g. gym, community allotment)

2. Diet and lifestyle behaviours

- **Eating habits before diagnosis**
  - fruit and veg, dairy, meat, high fat/sugar
  - alcoholic drinks, juice
  - supplements, herbal products, others
- **Remained the same**?

IF NO

- **Describe changes**
  - adding/removing/change dietary pattern (overhaul)
- **When** change
- **Reasons** for change
  - perception of diet before diagnosis
  - beliefs (role of diet in prostate cancer)
  - social and/or family pressure

For peer review only - http://bmjopen.bmj.com/site/about/guidelines.xhtml

- **Reasons** for change *(continued)*
  - control
  - fear of recurrence/improve overall wellbeing
- **Maintenance**

- **Motivators and facilitators**
  - wife/partner/family (food purchase and cooking)
  - advice, information (advice from Health Professionals)
  - benefits to health/prostate cancer

- **Barriers (especially for maintaining changes)**
  - cost, time
  - risk to health/prostate cancer
  - treatment side-effects/fatigue/general health

- **Physical activity/exercise before diagnosis**
  - Types
  - how often, duration
  - where (gym, local leisure centre, parks)
- **Remained the same**?

IF NO
- Describe changes
- When change
- Reasons for change

NB: For PA, similar questions on motivators/facilitators, and barriers.

3. Information provision

- **Understanding and awareness**
  - explore what healthy eating means
  - know of any healthy eating/PA advice (source of advice)

- **Information about diet and lifestyle**
  - sources
  - views on information

- **Preferences**
  - what type of information
  - who should provide information (health professionals)
  - what format

For peer review only - http://bmjopen.bmj.com/site/about/guidelines.xhtml

University of BRISTOL

University Hospitals Bristol NHS
NHS Foundation Trust

4. Experience and views of dietary and lifestyle intervention

- **Participate in research study** - asked to change your **eating habits**

If YES

- where/how receive information
- types (delivery format)
- duration
- reasons for participating

***If NO,* why not? How to promote participation?**

- **Participate in research study** - asked to change your **physical activity**

If YES

- where/how receive information
- types (delivery format)
- duration
- reasons for participating

***If NO,* why not? How to promote participation?**

5. Preferences for intervention

- If we ask you to change your eating habits, for example cooked tomatoes, how would you feel about this? (soya products, fish, lycopene supplement)

If POSITIVE

- duration
- where/how receive information (leaflets, telephone)
- what would make it easier/harder
- when (after diagnosis/treatment)

***If NEGATIVE,* why? What would make it easier? Different type of intervention?**

- If we ask you to walk extra 30mins everyday, how would you feel about this?

If POSITIVE

- duration, type (brisk walking/stroll)
- where/how receive information (leaflets, telephone)
- what would make it easier/harder
- when (after diagnosis/treatment)

***If NEGATIVE,* why not? What would make it easier? Different type of intervention?**

For peer review only - http://bmjopen.bmj.com/site/about/guidelines.xhtml

Closing

- Thank you
- Any questions?
- Summary of results from the study?
- £15 voucher (thank you for your time)

*This topic guide was amended during data collection to include additional questions on concerns about body weight, familiarity with soya products and tomatoes as a feature of a traditional Caribbean diet. They were identified as salient topics in interviews with the first few participants.

For peer review only

For peer review only - http://bmjopen.bmj.com/site/about/guidelines.xhtml

Supplementary Material 2: Additional quotes from interviews

**Theme 1: Pre-cancer diet and lifestyle**	
Men who perceived themselves to have a healthy diet before prostate cancer did not change their diet	I don't, I don't make no changing. But as I says, I don't overdo things…I normally look for things what is fat free…That's why I, I eat, if I eat bread I eat brown bread. And if I drink tea with, tea with milk I will drink the green top. (Jamal)
The proposed dietary intervention-increasing tomato intake-was acceptable to men as they are commonly consumed as part of a traditional Caribbean diet	"Yeah, we cook with a lot of tomatoes. I cook it. That's part of what we call the seasonal, you know, tomato, onions and thyme, garlic…So tomato is in everything and the seasoning. (Thomas)
Men who perceived themselves to be physically active before prostate cancer did not think they should be more active, and advocated moderation	… I don't run marathons, (Laugh) you know, I never did that, you know…maybe I could do a bit more [walking], but I don't. And at the moment I don't think I would, you know. Because I consider myself, I do keep fairly active. (Jonah)
	Well I, I exercise at the allotment (Laugh) as much as possible…So we're active, you know, may- maybe not, no one is pushing you. What we, we do a little and I sit down, and don't push yourself… (Colton)
Pre-existing injuries or health conditions as a barrier to physical activity	"…I used to do karate for 20 something years but, er, I have an injury so I give it up" (Albert).
	Just walking, yes. Because, like, I don't want to do too much because of like, my asthma... (Shaun)
**Theme 2: Evidence: link between diet, lifestyle, and prostate cancer**	
Men did not think prostate cancer is linked to diet and lifestyle as they perceived themselves to have a healthy diet and lifestyle	I don't think it has anything to do with it. That's my view. I mean, you might differ. But I don't think it has anything to do with my diet. Because I think I eat all the right stuff. (Matthew)
Conflicting dietary messages	Er, I took cod liver capsule, I stopped because it's no good after the time, they say they find out it's no good for you, for, because, for long term they can give you prostate cancer too…Yeah. So if you listen, you know, [some of this diet] everything you do is no good, you know, so you eat nothing. (Albert)
Clinicians uncertain about the effectiveness of diet/lifestyle	he [doctor] can't give us any direction or any what to do at the moment, because they did say they're not sure… So they are not sure yet, so obviously

For peer review only

on delaying or preventing prostate cancer progression	they can't give you any advice. All they are trying is to keep fit, you know, eat healthy and things like that. (Dennis)
**Theme 3: Coping with prostate cancer: Just get on with it**	
Men who wanted to return to a 'normal' life after prostate cancer were less likely to change or seek dietary/lifestyle information	Well really, to be honest, I never accept, I knew the idea of prostate leads to cancer, but I never thought that I had got it, I had it. I never think, "Oh I've got cancer." Nothing like that, no…but I was lucky enough I, I took it at the early stage, so it didn't have to develop… Just carry on with my normal life, you know. Do everything in moderation. (Fabian)
Social network and support as a facilitator to dietary and physical activity changes	…She's [sister] telling me that, you know, sugar and dairy products, that's the other things I must stop eating them things. (Laugh)...Since she told me that, I normally use skimmed milk to make my tea and I make porridge… I just try and do what she said. Less sugar, less dairy products. I just try to eat less of those things. I don't know. (Joseph)
	And it's just being in a room, with other people, that are trying to do something. There's a positive energy about being in a room… it's always better when you- well, when you walk in a pack, with people that are motivated the same way that you are. (Thomas)
**Theme 4: Ageing: Those were the days, this is me now**	
Ageing as a barrier to strenuous physical activity; adapting to changes to the body	Yeah I still do a lot of activities. Decorator, painting and decorating. I can do all that, but it's only now I, the ladder, my body is saying to me, "Don't climb the ladder much anymore."…suddenly you find the aches and pains and I can't climb the ladder anymore. (Fabian)
	Age. Cricket I am, I think last I played cricket was about maybe 15, 20 years ago and you feel as you get older you're restricted. I can't see myself running here and running there to catch a bus and that's not good for the heart. (Errol)
Ageing heightened men's awareness of their health, especially in regards to their body weight	… You know, it's a wise thing to change your diet, and I think I am reaching a certain age, you know… I think I should sacrifice and get rid of those things [starchy foods] and try and keep myself on a balanced diet where I could then chop my weight and look after myself… (Dennis)

For peer review only

	….I want to go to the gym soon…they reckon I'm putting on a bit too much weight so go and take off some of the weight (Laugh). (Sebastian)
**Theme 5: Autonomy: It has to be something I enjoy**	
Men who made changes out of necessity-chronic health conditions- broke their diet as they felt restricted	…but after you're diabetic you say okay, that's not good that's not good so you leave that out… Yes, so, so what I don't eating, I try to follow the rules as much as I can, but rules are meant to be broken. (Laugh). If we, if you obey all the rules all the time you miss half the fun. (Laugh) (Errol)
Preference in regards to incorporating tomatoes into diet	I eat tomatoes, but I don't like cooked ones…Fresh ones, I buy loads of them and I just squash them, cut them up and just eat them in my, eat them in bread and thing with my dinner. (Joseph)
Views on a dairy-free diet	Cut out the skimmed milk from, from my cereal, but I like my cereal, every morning. That would be difficult. I have my cereal every morning. I could cut out the cheese, but the milk. That's a difficult one for me. (Matthew)
Have to like the food to eat it, regardless of its benefits	I don't like, I can't eat the English type food most most 'cause a lot of things I don't I never eat it. They say it's good to eat, and I never eat it… (Joseph)
	…but it must be, not all fruit is palatable to me. I, I'm, I'm must like it to eat it, whether it's good for me or not I must like it. (Errol)

For peer review only

COREQ (COnsolidated criteria for REporting Qualitative research) Checklist

A checklist of items that should be included in reports of qualitative research. You must report the page number in your manuscript where you consider each of the items listed in this checklist. If you have not included this information, either revise your manuscript accordingly before submitting or note N/A.

Topic	Item No.	Guide Questions/Description	Reported on Page No.
**Domain 1: Research team and reflexivity**			
Personal characteristics			
Interviewer/facilitator	1	Which author/s conducted the interview or focus group?	P8, L2
Credentials	2	What were the researcher's credentials? E.g. PhD, MD	P8, L7
Occupation	3	What was their occupation at the time of the study?	P8, L6
Gender	4	Was the researcher male or female?	P8, L6
Experience and training	5	What experience or training did the researcher have?	P8, L7-8
Relationship with participants			
Relationship established	6	Was a relationship established prior to study commencement?	No
Participant knowledge of the interviewer	7	What did the participants know about the researcher? e.g. personal goals, reasons for doing the research	P8, L6-7
Interviewer characteristics	8	What characteristics were reported about the inter viewer/facilitator? e.g. Bias, assumptions, reasons and interests in the research topic	P8, L8-12
**Domain 2: Study design**			
Theoretical framework			
Methodological orientation and Theory	9	What methodological orientation was stated to underpin the study? e.g. grounded theory, discourse analysis, ethnography, phenomenology, content analysis	P9, L4
Participant selection			
Sampling	10	How were participants selected? e.g. purposive, convenience, consecutive, snowball	P7, L11-17
Method of approach	11	How were participants approached? e.g. face-to-face, telephone, mail, email	P7, L9-11
Sample size	12	How many participants were in the study?	P7, L21
Non-participation	13	How many people refused to participate or dropped out? Reasons?	We did not rec
Setting			
Setting of data collection	14	Where was the data collected? e.g. home, clinic, workplace	P8, L2-4
Presence of non-participants	15	Was anyone else present besides the participants and researchers?	No
Description of sample	16	What are the important characteristics of the sample? e.g. demographic data, date	P9, L18-23; Tab
Data collection			
Interview guide	17	Were questions, prompts, guides provided by the authors? Was it pilot tested?	P8, L14-22; Sup
Repeat interviews	18	Were repeat inter views carried out? If yes, how many?	No
Audio/visual recording	19	Did the research use audio or visual recording to collect the data?	P8, L1-2
Field notes	20	Were field notes made during and/or after the inter view or focus group?	
Duration	21	What was the duration of the inter views or focus group?	P8, L
Data saturation	22	Was data saturation discussed?	P7, L20-22
Transcripts returned	23	Were transcripts returned to participants for comment and/or	No

For peer review only

Topic	Item No.	Guide Questions/Description	Reported on Page No.
		correction?	
**Domain 3: analysis and findings**			
Data analysis			
Number of data coders	24	How many data coders coded the data?	P9, L5-8
Description of the coding tree	25	Did authors provide a description of the coding tree?	N/A
Derivation of themes	26	Were themes identified in advance or derived from the data?	P9, L5-7
Software	27	What software, if applicable, was used to manage the data?	
Participant checking	28	Did participants provide feedback on the findings?	No
Reporting			
Quotations presented	29	Were participant quotations presented to illustrate the themes/findings? Was each quotation identified? e.g. participant number	Results, P10-18
Data and findings consistent	30	Was there consistency between the data presented and the findings?	P9, L5-12; L14-[illegible]
Clarity of major themes	31	Were major themes clearly presented in the findings?	Results, P10-18
Clarity of minor themes	32	Is there a description of diverse cases or discussion of minor themes?	Discussion, P1[illegible]

Developed from: Tong A, Sainsbury P, Craig J. Consolidated criteria for reporting qualitative research (COREQ): a 32-item checklist for interviews and focus groups. *International Journal for Quality in Health Care*. 2007. Volume 19, Number 6: pp. 349 – 357

Once you have completed this checklist, please save a copy and upload it as part of your submission. DO NOT include this checklist as part of the main manuscript document. It must be uploaded as a separate file.

For peer review only
